# The Splice Index as a prognostic biomarker of strength and function in myotonic dystrophy type 1

**DOI:** 10.1172/JCI185426

**Published:** 2025-01-07

**Authors:** Marina Provenzano, Kobe Ikegami, Kameron Bates, Alison Gaynor, Julia M. Hartman, Aileen Jones, Amanda Butler, Kiera N. Berggren, Jeanne Dekdebrun, Man Hung, Dana M. Lapato, Michael Kiefer, Charles A. Thornton, Nicholas E. Johnson, Melissa A. Hale, on behalf of the Myotonic Dystrophy Clinical Research Network (DMCRN)

**Affiliations:** 1 Center for Inherited Myology Research and; 2 Department of Neurology, School of Medicine, Virginia Commonwealth University, Richmond, Virginia, USA.; 3 Children’s Hospital of Richmond at Virginia Commonwealth University, Pediatric Therapy Services, Richmond, Virginia, USA.; 4 Department of Neurology, University of Rochester School of Medicine and Dentistry, Rochester, New York, USA.; 5 College of Dental Medicine, Roseman University of Health Sciences, South Jordan, Utah, USA.; 6 Department of Orthopaedics, University of Utah, Salt Lake City, Utah, USA.; 7 Department of Human and Molecular Genetics, School of Medicine and; 8 Department of Physical Therapy, College of Health Professions, Virginia Commonwealth University, Richmond, Virginia, USA.; 9 Center for RNA Biology, University of Rochester School of Medicine and Dentistry, Rochester, New York, USA.; 10 The DMCRN consortium is detailed in Supplemental Acknowledgments.

**Keywords:** Muscle biology, Neuromuscular disease, RNA processing, Skeletal muscle

## Abstract

**BACKGROUND:**

Myotonic dystrophy type 1 (DM1) is a multisystemic, CTG repeat expansion disorder characterized by a slow, progressive decline in skeletal muscle function. A biomarker correlating RNA mis-splicing, the core pathogenic disease mechanism, and muscle performance is crucial for assessing response to disease-modifying interventions. We evaluated the Myotonic Dystrophy Splice Index (SI), a composite RNA splicing biomarker incorporating 22 disease-specific events, as a potential biomarker of DM1 muscle weakness.

**METHODS:**

Total RNA sequencing of tibialis anterior biopsies from 58 DM1 participants and 33 unaffected/disease controls was used to evaluate RNA splicing events across the disease spectrum. Targeted RNA sequencing was used to derive the SI from biopsies collected at baseline (*n* = 52) or a 3-month (*n* = 37) follow-up visit along with clinical measures of muscle performance.

**RESULTS:**

The SI demonstrated significant associations with measures of muscle strength and ambulation, including ankle dorsiflexion (ADF) strength and 10-meter run/fast walk (Pearson’s *r* = –0.719 and –0.680, respectively). The SI was relatively stable over 3 months (intraclass correlation coefficient [ICC] = 0.863). Latent-class analysis identified 3 DM1 subgroups stratified by baseline SI (SI_Mild_, SI_Moderate_, and SI_Severe_); SI_Moderate_ individuals had a significant increase in the SI over 3 months. Multiple linear regression modeling revealed that baseline ADF and SI were predictive of strength at 3 months (adjusted *R*² = 0.830).

**CONCLUSION:**

The SI is a reliable biomarker that captures associations of RNA mis-splicing with physical strength and mobility and has prognostic utility to predict future function, establishing it as a potential biomarker for assessment of therapeutic target engagement.

**TRIAL REGISTRATION:**

ClinicalTrials.gov NCT03981575.

**FUNDING:**

FDA (7R01FD006071), Myotonic Dystrophy Foundation, Wyck Foundation, Muscular Dystrophy Association, Novartis, Dyne, Avidity, PepGen, Takeda, Sanofi Genzyme, Pfizer, Arthex, and Vertex Pharmaceuticals.

## Abstract 2

The Myotonic Dystrophy Splicing Index is a biomarker that correlates the primary disease mechanism with measures of skeletal muscle performance in affected individuals.

## Graphical abstract

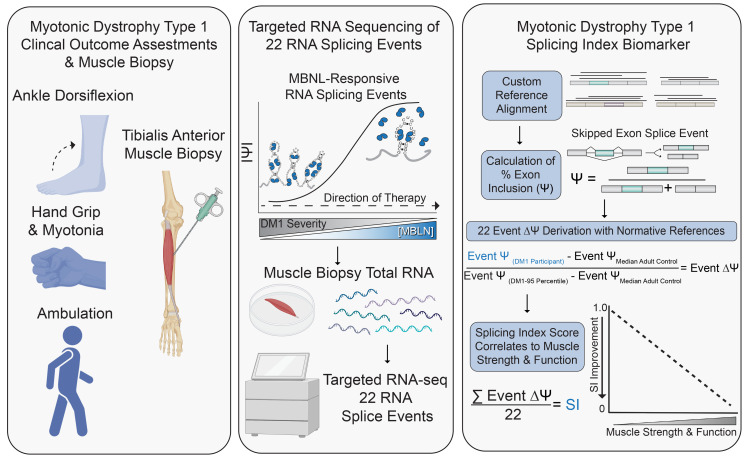

## Introduction

Myotonic dystrophy type 1 (DM1) is the most common form of adult-onset muscular dystrophy, with an estimated gene frequency of 1:2100 (1). This autosomal dominantly inherited disorder is characterized by a core triad of myotonia (i.e., difficulty relaxing muscles after contraction), progressive distal muscle weakness, and early-onset cataracts. Beyond these characteristic symptoms, DM1 is a multisystemic disorder affecting nearly every organ in the body, including the CNS, GI tract, and cardiovascular system (2). The impact on these organ systems results in the presentation of daytime sleepiness, cardiac arrhythmias, and respiratory failure. This complex presentation leads to a phenotypically diverse population with variable symptomatic presentation and progression.

DM1 is caused by an expanded CTG repeat tract [(CUG)_exp_] in the 3′ untranslated region of the *DMPK* gene (3–5). (CUG)_exp_ RNAs transcribed from this locus are toxic due to their propensity to sequester RNA binding proteins, most notably muscleblind-like (MBNL) (6–8). This alternative splicing factor is a key driver of fetal to adult RNA isoform transitions during development and its sequestration leads to widespread dysregulation of alternative splicing and retention of mRNA transcripts encoding nonfunctional fetal isoforms of the associated gene products (9–13). Mis-splicing of select MBNL-dependent events have been linked to disease symptoms, including insulin resistance (*INSR*), myotonia (*CLCN1*), and cardiac arrhythmia (*SCN5A*) in DM1 cell and mouse models (14–21). Prior work has demonstrated that these are titratable events whereby a dose-response relationship is observed between changes in RNA splicing and the relative sequestration of MBNL1 (22, 23). This dose-dependent relationship is event specific, whereby individual RNA splicing events are more or less sensitive to functional concentrations of MBNL associated with the spectrum of DM1 disease severity (22, 23). In concordance with these observations, RNA splicing events are differentially rescued in DM1 preclinical models across a therapeutic dose range and have been shown to differentially respond to interventional knockdown of the *DMPK* transcript in a human clinical trial (24–29). These findings have been mirrored in evaluation of mis-splicing within a group of DM1 individuals where select events had stronger or weaker correlations with manual muscle testing of the ankle dorsiflexion (ADF) (30).

Given its relevance to disease pathology and correlation with physical function in DM1, RNA splicing of MBNL-dependent, disease-associated events is a promising biomarker for preclinical and clinical evaluation of therapeutic engagement. However, in isolation, no single event has been shown to capture the range of molecular and phenotypic variability observed in DM1 individuals, making it difficult to match the degree of mis-splicing of an isolated RNA event to the status of overall physical function in an individual (30). Previous work has sought to develop a composite splicing measure that could capture the range of phenotypic severity observed. In DM1 mouse models, a composite measure of 35 splicing events showed a graded response to decrements of Mbnl or increasing (CUG)_exp_ load (28). Early efforts to identify human candidate biomarkers utilized a measure known as [MBNL]_inferred_, which estimates intracellular levels of free, functional MBNL using a small collection of RNA splicing events highly predictive of global mis-splicing changes in DM1 tibialis anterior (TA) muscle (22, 31). However, these studies are constrained by limited availability of skeletal muscle biospecimens from a large, well-characterized cohort inclusive of longitudinal sampling to comprehensively profile the complete spectrum of RNA mis-splicing and assess temporal changes over the course of disease progression.

Here, we generated a total RNA sequencing (RNA-seq) dataset encompassing 95 skeletal muscle transcriptomes from an extensively phenotyped cohort of 58 DM1 participants, a subset of whom provided longitudinal biopsies at baseline (BL) and a 3-month (3M) follow-up visit. This dataset was utilized to assess the validity of a composite RNA splicing biomarker called the Myotonic Dystrophy Splice Index (SI). Leveraging high-resolution, targeted amplicon RNA-seq of a 22-splice-event panel, we demonstrate that the SI accurately assesses and normalizes the relative degree of skeletal muscle splicing dysregulation across the DM1 disease spectrum, is dynamic and sensitive to changes over time, and displays strong correlations with multiple measures of concurrent and future muscle performance. We also evaluate the ability of the SI to stratify mild, moderate, and severe cases of DM1 and leverage our longitudinally sampled subcohort to assess the utility of this biomarker to predict changes in physical function over time. This molecular marker provides a useful tool to capture the spectrum of DM1 disease severity and may serve as a biomarker of target engagement in clinical trials.

## Results

### Phenotypically characterized DM1 participant cohort displays heterogeneous global splicing dysregulation across the spectrum of disease severity.

**Figure 1:**
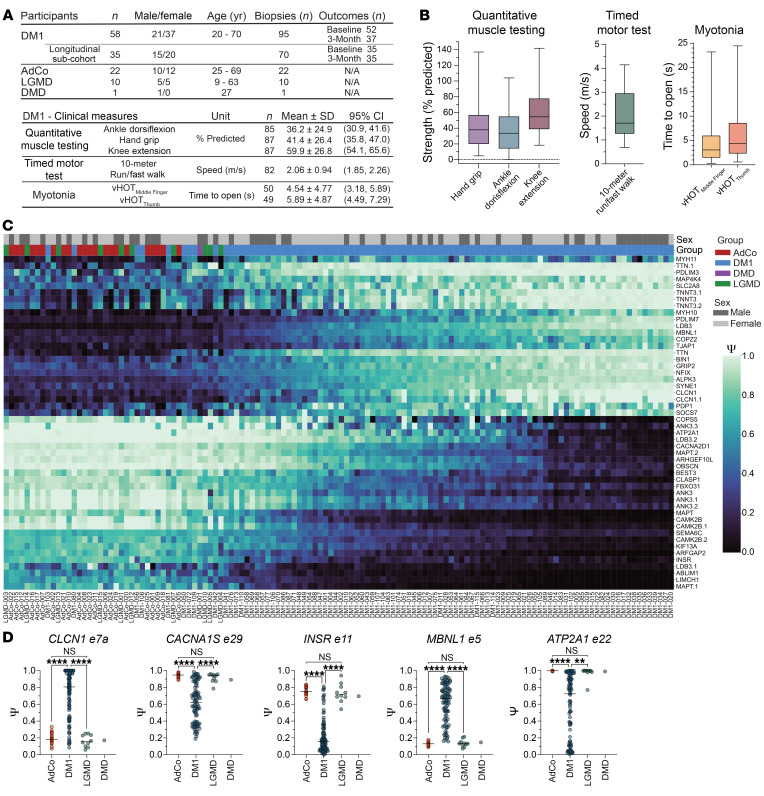
DM1 participant cohort displays broad spectrum of RNA splicing dysregulation, as assessed by total RNA-seq. (**A**) DM1 participant demographic information, including sample size, age at biopsy, and sex distribution in complete cross-sectional and longitudinal cohort subset. The mean normalized performance on clinical outcome measures for the cross-sectional DM1 cohort are reported along with sample size, mean ± SD, and 95% CI. Additional demographic information of all samples used in this report are provided in Supplemental Table 1. (**B**) Box-and-whisker plots of outcome measure performance in DM1 cross-sectional cohort, where the lines represents median performance, the bounds of the boxes are the 25th and 75th percentiles, and whiskers extend to maximum and minimum values. (**C**) Heatmap displaying estimated percentage spliced (Ψ) of top 50 significantly dysregulated skipped exon (SE) events between DM1 versus unaffected adult controls (AdCo) and disease control reference groups (DMD and LGMD) subjected to total RNA-seq (|ΔΨ| ≥ 0.1, FDR ≤ 0.05). Both rows (SE events) and columns (individual samples) were subjected to hierarchical clustering. Sample group and sex are annotated above the heatmap and individual IDs are reported below. (**D**) Ψ values for specific SE events in all sample groups (*n* = 22 AdCo, *n* = 95 DM1, *n* = 10 LGMD, and *n* = 1 DMD). Bar represents median. **P < 0.01; ****P < 0.0001. NS, not significant. One-way ANOVA with Tukey’s correction, where DMD was not included in the analysis due to insufficient sample size.

To advance DM1 biomarker development, we assembled a cohort of 58 adult-onset DM1 participants aged 21 to 70 years (Figure 1A and Supplemental Table 1; supplemental material available online with this article; https://doi.org/10.1172/JCI185426DS1). This cohort included a total of 95 TA biopsies, with 35 individuals providing both BL and 3M longitudinal samples accompanied by time point–matched assessments of muscle performance. Cross-sectionally, this cohort displayed a wide range of muscle strength and performance, as measured by quantitative muscle testing (QMT) and timed motor tests (TMTs) (Figure 1B). Variability in the degree of myotonia (the inability to relax muscles after contraction) was also evident, as measured by the video hand opening time (vHOT) (Figure 1B and Supplemental Figure 1A).

To obtain a comprehensive view of RNA mis-splicing, we performed total RNA-seq on all available DM1 samples (*n* = 95) and evaluated global RNA splicing dysregulation. Additional muscle biopsies were included as reference groups from unaffected adults (AdCo, *n* = 22) and individuals with muscular dystrophies whereby the pathogenic mechanism is not predicted to cause RNA mis-splicing, including Duchenne muscular dystrophy (DMD) and limb-girdle (LGMD) types R1/2A, R3/2D, R7/2G, and R12/2L (*n* = 11) (Supplemental Table 1) (32–36). While clinical outcomes were unavailable for these unaffected and disease-control subgroups, their inclusion allowed for assessment of relative splicing dysregulation across the assembled DM1 cohort and disease specificity of the observed mis-splicing. Inclusion levels (Ψ) for skipped exon (SE) splicing events were calculated via comparison of all DM1 individuals versus unaffected individuals and disease controls.

Principal component analysis of significantly mis-spliced events (|ΔΨ| ≥ 0.1, FDR ≤ 0.05, *n* = 946 SE events) illustrated the expected distribution; while unaffected individuals clustered tightly, all DM samples were distributed evenly across the first principal component consistent with the well-established spectrum of splicing dysregulation observed in other studies (Supplemental Figure 1B) (22, 31). The heterogeneity of mis-splicing across the DM1 cohort is further demonstrated by visualization of the top 50 most differentially spliced events (Figure 1C and Supplemental Table 2). While controls displayed minimal variability in Ψ, DM1 individuals exhibited the full spectrum of splicing dysregulation, ranging from splicing patterns resembling AdCo to those with maximal mis-splicing. Disease controls clustered tightly with AdCo samples, consistent with the predicted lack of significant RNA mis-splicing in these muscular dystrophy subtypes (Figure 1C and Supplemental Figure 1B) (32–36).

The breadth of splicing dysregulation within the DM1 cohort is further demonstrated via visualization of select disease-specific events, including *CLCN1* exon 7a (e7a), *CACNA1S* e29, *INSR* e11, *ATP2A1* e22, and *MBNL1* e5 (Figure 1D) (14–18, 20, 37–39). Collectively, this DM1 cohort captures a comprehensive spectrum of disease-specific, global splicing dysregulation and a diverse range of skeletal muscle phenotypes. These features establish this dataset as a robust resource for biomarker assessment, offering a representative snapshot of the range of molecular pathology underlying DM1.

### Selection and evaluation of splicing events for inclusion in a composite RNA splicing biomarker panel.

While total RNA-seq is a powerful discovery tool for comprehensively capturing and describing global splicing dysregulation in DM1, ultra-deep sequencing coverage is required for precise quantification of exon inclusion. Computational estimation of Ψ relies on sequence assembly and adequate splice-site junction coverage. As such, short paired-end sequencing commonly utilized for transcriptomic analysis has reduced power to detect target splicing events in low-abundance transcripts or those with complex splice site utilization. If a subset of splicing events can distill patterns of global splicing dysregulation, targeted amplicon RNA-seq with extended read lengths may offer a more efficient and sensitive tool to detect RNA splicing changes. Previously, total RNA-seq identified 35 candidate splicing events for a targeted RNA-seq panel aimed at developing a species-specific composite splicing metric in DM1 mouse models (28).

Building on these past efforts, we leveraged our DM1 cohort to evaluate whether a defined set of RNA splicing events can adequately capture the full range of DM1 splicing dysregulation, especially as prior statistical modeling indicates improved accuracy in estimating changes in MBNL activity using between 20 and 30 events (22). In total, a combination of 22 events were identified for inclusion based on the following criteria: (a) DM1-specific mis-splicing, (b) pronounced and widespread changes in Ψ within DM1 samples, (c) prior evaluation as biomarkers of muscle strength, and (d) collectively spanning a broad range of sensitivity to estimated MBNL levels (29, 30).

**Figure 2:**
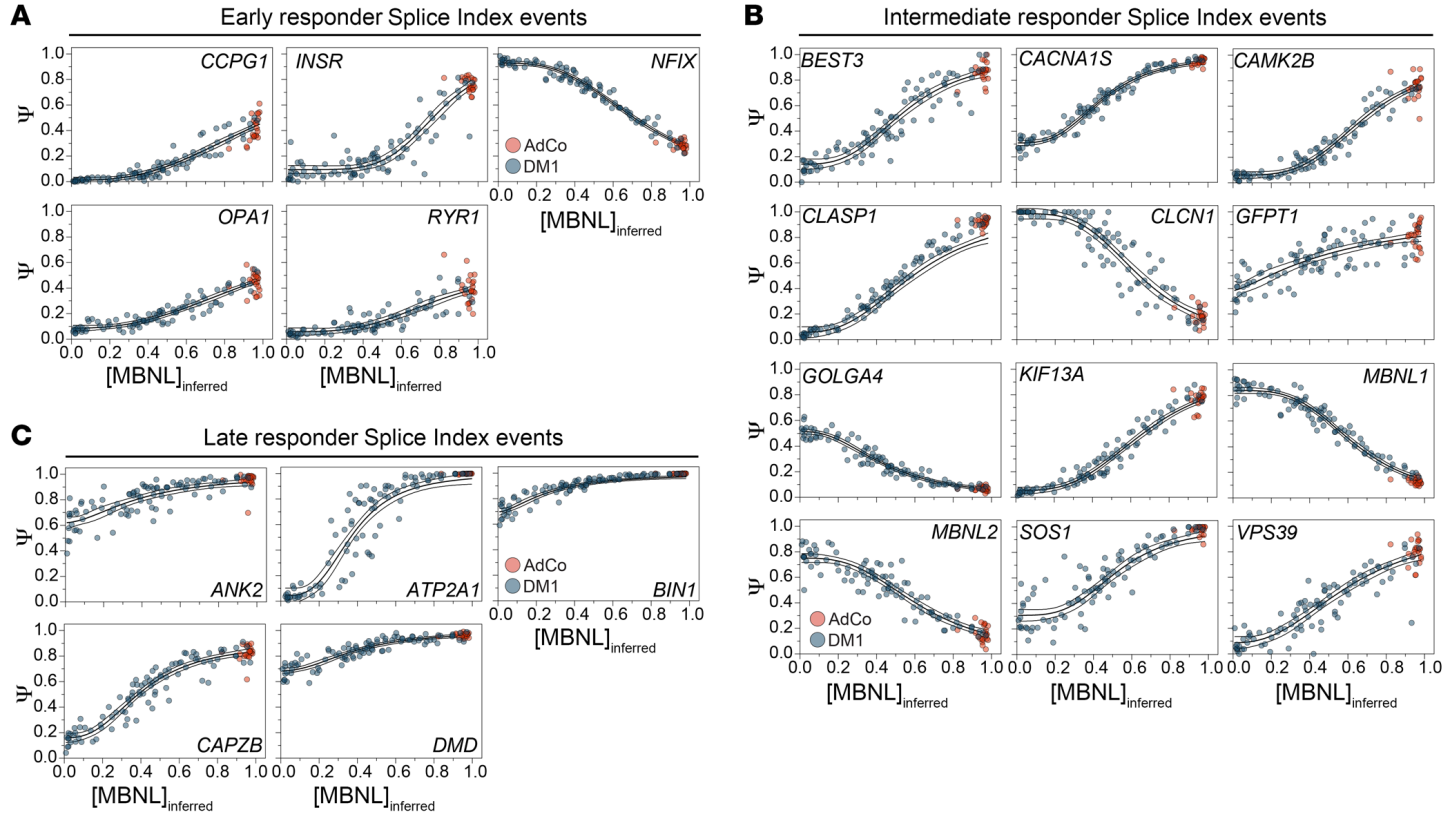
RNA splicing events included in composite Splice Index capture RNA mis-splicing patterns observed across the range of estimated, functional MBNL concentrations in DM1 cohort. (**A**–**C**) Twenty-two RNA splicing events included in the Splice Index (SI) panel demonstrate variable sensitivity to [MBNL] observed across the spectrum of DM1 participants. Dose-response curves of 22 events included in the composite SI. Ψ values derived from total RNA-seq (Supplemental Table 3) were plotted against estimated functional concentrations of MBNL ([MBNL]_inferred_). Ψ values from both unaffected adult controls (AdCo, *n* = 22) and DM1 participants (*n* = 95) were fit to a 4-parameter dose-response curve and events classified as (**A**) early, (**B**) intermediate, and (**C**) late responder events based on estimated EC_50_ relative to the observed median of all events. Intermediate responder events were classified based on EC_50_ values within the interquartile range of 22-event distribution. Early responder were classified based on an estimated EC_50_ in the 25th percentile, while late responder events had values in the 75th percentile. Curve fit is annotated as the thick solid line and the 95% CI of the fit is shown as the above and below lines. All curve-fit parameters are reported in Supplemental Table 4.

All 22 events exhibited strong DM1-specific mis-splicing compared with unaffected AdCo and LGMD/DMD disease controls as well as a wide distribution of exon inclusion across DM1 participants (Supplemental Figure 2). Given our aim to detect Ψ shifts across the full spectrum of DM1 spliceopathy, Ψ values derived from total RNA-seq were plotted against estimated free MBNL protein concentrations ([MBNL]_inferred_) (22). Each event was then fit to a 4-parameter dose-response curve to quantify parameters reflective of biological phenomena, including an EC_50_ value representative of the relative midpoint of the [MBNL] dose response (22, 23). These EC_50_ values were used to identify and classify events based on their sensitivity to changes in free [MBNL] as early, intermediate, and late responder events (i.e., high, medium, and low [MBNL]) (Figure 2, A–C).

Approximately half of the 22 selected events (*n* = 12) demonstrated stepwise changes in Ψ across the [MBNL]_inferred_ gradient, with intermediate EC_50_ values near the median of the complete panel (median EC_50_ = 0.619) (Supplemental Table 4). These intermediate responder events, classified by EC_50_ values within the interquartile range of the panel, accurately capture the largest proportion of splicing dysregulation within the DM1 cohort (Figure 2B). To ensure sensitive detection of RNA splicing changes in individuals with mild and severe spliceopathy, early and late responder events, respectively, were also identified and incorporated. Early responder events (*n* = 5 of 22) were mis-spliced upon a small reduction in free [MBNL] (EC_50_ = 0.745–1.03) (Figure 2A). Conversely, late responder events (*n* = 5 of 22) exhibited limited sensitivity to changes in free [MBNL], whereby mis-splicing was observed only in those participants with the most severe spliceopathy (EC_50_ = 0.390–0.263) (Figure 2C).

### Composite SI robustly and reliably captures global RNA splicing dysregulation using targeted RNA-seq.

We next sought to evaluate whether the 22-RNA splicing event panel could effectively capture DM1 mis-splicing using a targeted, amplicon-based RNA-seq methodology similar to that previously described for use in a DM1 mouse model (28). Multiplex RT-PCR was performed with a low RNA input followed by targeted amplicon RNA-seq of event inclusion and exclusion products (*n* = 95 DM1 and 22 AdCo). As detection bias is a potential disadvantage of this approach due to preferential amplification and sequencing of smaller, exon exclusion isoforms, we compared Ψ values between total and targeted RNA-seq datasets. Consistent with this expectation, targeted RNA-seq Ψ estimates were generally lower, but not all splicing events were impacted (Supplemental Figure 3A). The mean underdetection bias was approximately 7%, with greater bias observed for events with longer cassette exons. The 2 events with the largest cassette exons (*KIF13A* at 120 nt and *NFIX* at 123 nt) showed the strongest underdetection of exon inclusion at 19% and 15%, respectively. Conversely, almost no underdetection was observed for 2 of the smallest exons within the panel (*RYR1* at 15 nt and *VPS39* at 33 nt) (Supplemental Figure 3B). Despite this limitation of amplicon sequencing, the greater uniformity of read coverage across relevant splice junctions compared to total RNA-seq, reduced confidence limits for Ψ estimates, and significantly higher read counts per splicing event yield other valuable advantages. Taken together, these results support the feasibility of targeted RNA-seq as an approach to accurately capture Ψ of select splicing events using low RNA inputs from muscle biopsies.

To summarize the extent of splicing dysregulation captured by all 22 events in DM1 participants, we developed a composite metric termed the Myotonic Dystrophy Splicing Index (SI). To derive an individual SI score, Ψ values were first normalized to standardized reference values to scale each individual Ψ within boundaries of splicing dysregulation observed between unaffected, healthy controls (Ψ_Median Control_) and the most severely affected DM1 participants (Ψ_DM95_, 95th percentile within the DM1 sample distribution). The complete spectrum of normalized Ψ values observed within our collective sample group can be visualized via heatmap (Supplemental Figure 4A). Normalized Ψ values were averaged to generate a value from 0 to 1, where values closer to 1 represent the most severe mis-splicing. Normative reference Ψ values for all 22 events were established using all samples subjected to targeted RNA-seq (*n* = 22 AdCo and 95 DM1) (Supplemental Table 6). These reference values showed strong agreement with those derived from secondary, non-overlapping AdCo and DM1 cohorts (*n* = 172 DM1 and 25 AdCo, Ψ_DM95_ intraclass correlation coefficient [ICC] = 0.996, Ψ_Median Control_ ICC = 0.996, *P* < 0.0001) and SI scores derived using either normative reference set were highly concordant (Supplemental Figure 4B) (29).

Consistent with the range of splicing dysregulation observed via total RNA-seq, SI scores for DM1 participants spanned the full range (SI = 0.0–1.0), while AdCo samples exhibited consistently low SI scores (SI = 0.0–0.129) (Supplemental Figure 4C). Technical replication of library preparation, sequencing, and SI calculation demonstrated a high degree of test-retest reliability (ICC = 0.999, *P* < 0.0001, *n* = 6 DM1 and 1 AdCo) (Supplemental Figure 4D) (22, 31). Principal component analysis of total RNA-seq data for 946 mis-spliced exons determined that 85% of the between-participant variance was defined by the first principal component (Supplemental Figure 1B). Targeted sequencing of SI events recapitulated nearly all between-participant splicing variance (*R*^2^ = 0.96), indicating that the 22 selected SI events effectively capture the disease-associated distribution of mis-splicing (Supplemental Figure 4, E and F). These findings establish the SI as a robust, reliable, and disease-specific measure of splicing dysregulation in DM1.

### The SI correlates strongly with measures of muscle strength and motor function.

**Figure 3:**
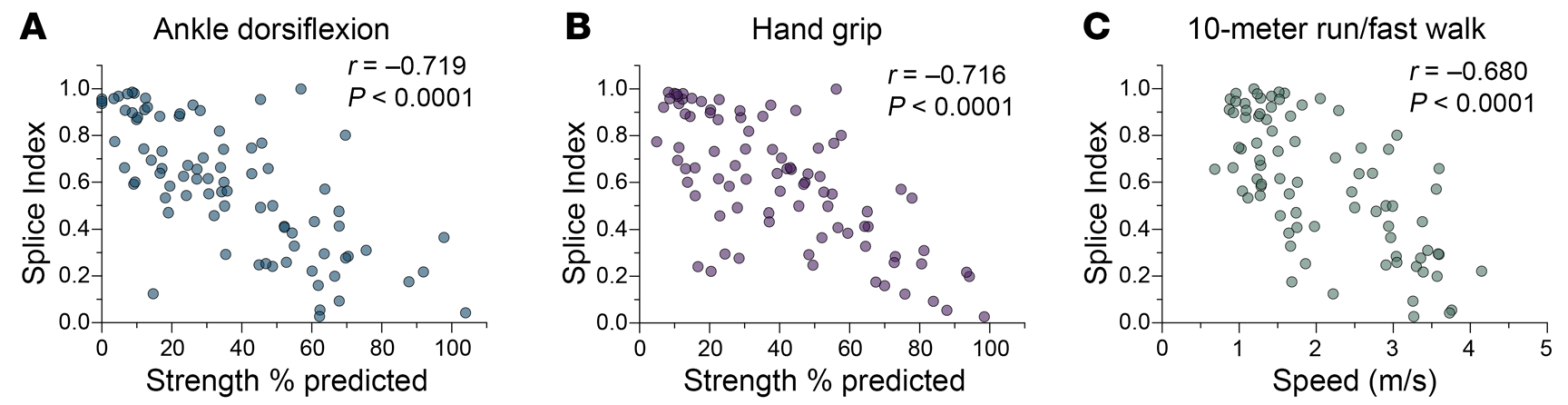
Splice Index correlates strongly with clinical outcome measures of muscle strength and motor function in cross-sectional DM1 cohort. (**A** and **B**) Correlation plots of Splice Index (SI) versus quantitative measures of ankle dorsiflexion (ADF) strength and hand grip strength (HGS), respectively. Individual measures are reported as the percentage of predicted strength compared with unaffected individuals. ADF Pearson’s *r* = –0.719 [–0.808, –0.597], *n* = 85 and HGS Pearson’s *r* = –0.716 [–0.805, –0.595], *n* = 87. (**C**) Correlation plot of SI versus 10-meter run/fast walk. Individual measures are reported as speed (meters/second). Pearson’s *r* = –0.680 [–0.782, –0.543], *n* = 82. All correlations are reported as Pearson’s *r* [95% CI] with 2-tailed *P* values.

To evaluate the SI as a biomarker of phenotypic disease severity, we assessed its correlations with skeletal muscle performance in DM1 participants with time point–matched functional outcome assessments. As DM1 predominantly affects distal muscles, we first evaluated strength in hand grip and ADF. Higher SI values were strongly correlated with weaker ADF and hand grip strength (HGS) (Pearson’s *r* = –0.719 and –0.716, respectively) (Figure 3, A and B). Knee extension (KE) showed a weaker correlation (Pearson’s *r* = –0.392), likely reflecting a disparity between proximal muscle strength and mis-splicing in the distal TA (Supplemental Figure 5A). The SI was also robustly correlated with motor function, as assessed by 10-meter run/fast walk speed (10MRW) (Pearson’s *r* = –0.680) (Figure 3C). In total, these findings indicate that the degree of mis-splicing as measured by the SI correlates with both physical strength and ambulation.

We further explored the association between myotonia and the SI. Multiple preclinical studies in DM1 mouse models have shown that myotonia rapidly and sensitively responds to administration of disease-modifying therapies that increase free [MBNL], making clinical measures of myotonia and their association with a molecular biomarker ideal for assessment of target engagement in forthcoming clinical trials (24, 25). The SI moderately correlated with vHOT, a measure of myotonia (vHOT_Middle Finger_ Spearman’s *r* = 0.454 and vHOT_Thumb_ Spearman’s *r* = 0.378) (Supplemental Figure 5, B and C). Individuals with high SI scores had extended times to open a closed fist, while those with minimal splicing dysregulation displayed low to no clinical myotonia. The reduced strength of these associations with the SI compared with other clinical assessments are more likely related to both the clinical variability of myotonia and the measurement imprecision of this assessment rather than the lack of biological association with the underlying pathophysiology. Overall, the SI demonstrated significant cross-sectional correlations with multiple functional outcome measures, underscoring the relationship between splicing dysregulation in DM1 skeletal muscle and measures commonly used to clinically evaluate DM1 disease severity.

While the complete composite SI displayed robust associations with phenotypic performance, we also examined correlations of normalized Ψ values from individual splicing events via multiple correlation analysis. Independent of outcome measure, all splicing events showed comparable or mildly reduced correlative power compared with the full composite SI, with most having absolute Pearson’s correlation coefficients greater than 0.5 for the most strongly correlated outcomes in our cross-sectional DM1 cohort (ADF, HGS, and 10MRW) (Supplemental Table 7). Distinct pattens emerged when ranking individual event correlations. Mis-spliced ion channels associated with defects in skeletal muscle excitation-contraction coupling and myotonia – *CACNA1S* e29 and *CLCN1* e7a – frequently ranked among the top 3 most significantly correlated events for ADF, HGS, and 10MRW (18, 20). These findings are consistent with our classification of these events as intermediate responders that show stepwise decrements in Ψ across the disease spectrum, enhancing their overall correlation with the majority of DM1 individuals’ performance. Conversely, events classified as early and late responder events, including *INSR* e11 and *BIN1* e11, displayed the weakest independent correlations (Supplemental Table 7).

### The SI sensitively captures longitudinal RNA splicing changes in DM1 skeletal muscle over 3M.

**Figure 4:**
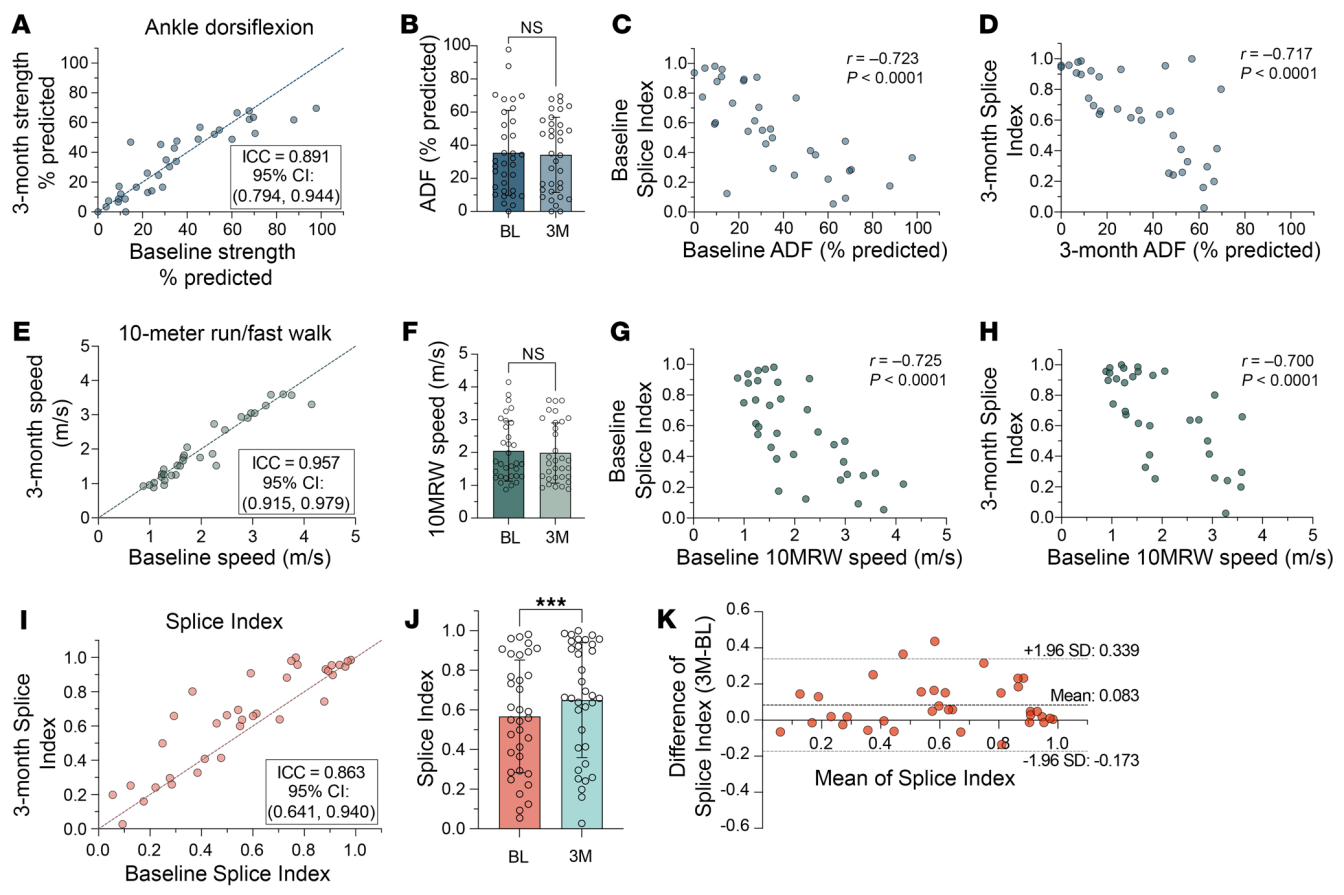
Mean Splice Index increases between baseline and 3 months in longitudinal DM1 cohort, with no changes in functional endpoints. (**A** and **B**) Assessment of changes in ankle dorsiflexion (ADF) strength in longitudinal DM1 cohort. (**A**) Comparison of baseline (BL) and 3-month (3M) ADF demonstrates strong test-retest reliability between time points. Line of agreement (*x* = *y*) is displayed and intraclass correlation coefficient (ICC) with 95% CI is reported. (**B**) Mean ADF strength is unchanged in longitudinal DM1 cohort at BL and 3M (*n* = 34). (**C** and **D**) BL (**C**) and 3M (**D**) Splice Index (SI) scores correlate strongly with time point–matched measures of ADF strength (*n* = 34). BL SI vs. BL ADF Pearson’s *r* = –0.723 [–0.852, –0.507] and 3M SI vs. 3M ADF Pearson’s *r* = –0.717 [–0.849, –0.499]. (**E** and **F**) Assessment of changes in 10-meter run/fast walk (10MRW) speed in longitudinal DM1 cohort. (**E**) Comparison of BL and 3M 10MRW speed demonstrates strong test-retest reliability (**F**) and no mean difference between time points (*n* = 32). (**G** and **H**) BL (**G**) and 3M (**H**) SI scores correlate strongly with time point–matched measures of 10MRW speed. BL SI vs. BL 10MRW, *n* = 34, Pearson’s *r* = –0.725 [–0.854, –0.513] and 3M SI vs. 3M 10MRW, *n* = 32, Pearson’s *r* = –0.700 [–0.843 –0.465]. (**I** and **J**) Assessment of changes in SI score over 3M in longitudinal DM1 cohort. (**I**) SI demonstrates moderate test-retest reliability (**J**), but a significant increase in mean SI score (*n* = 35). (**K**) Bland-Altman plot illustrating agreement between SI scores at BL and 3M in longitudinal subcohort. Dotted lines represent mean of the differences (bias) and 95% limits of agreement (mean of differences ± 1.96 SD). Data in **B**, **F**, and **J** are presented as mean ± SD and were analyzed with a paired, 2-tailed *t* test. ***P < 0.001. NS, not significant. All correlations reported as Pearson’s *r* [95% CI] with 2-tailed *P* values.

We next sought to evaluate the longitudinal stability of the SI by leveraging the inclusion of 35 DM1 participants in the cross-sectional cohort who provided longitudinal biopsies at BL and 3M follow-up visits (longitudinal subcohort, Figure 1A). Given the slow progression of DM1 with minimal declines in muscle strength and motor function observed over 1 year, we predicted that no significant changes in phenotypic presentation would be detected over this short time course (40–42). As expected, no significant differences were observed in clinical performance on outcome assessments, which demonstrated high test-retest reliability between BL and 3M (Figure 4, A, B, E, and F, and Supplemental Figure 6). SI scores remained strongly correlated with time point–matched functional assessments at comparable strength to the full cross-sectional cohort, despite the reduced sample size (Figure 4, C, D, G, and H, and Supplemental Figure 6).

Although functional endpoints did not change over this short time period, the mean SI was significantly increased between BL and 3M biopsies, indicative of a small increase in overall splicing dysregulation (Figure 4J). We initially inferred that this may be the consequence of biopsy type (14-gauge needle aspirate vs. Bergstrom) or leg selection (ipsilateral vs. contralateral) used for repeat biopsies. However, correlation analysis did not indicate that either variable related to muscle biopsy collection significantly influenced the SI, although sample sizes in some comparisons were small (Supplemental Figure 7). Consistent with the observed change in SI being representative of a true shift in splicing dysregulation within this cohort, test-retest reliability between BL and 3M SI scores was reduced (ICC = 0.863, *P* < 0.0001) compared with technical replicates of the same samples (Figure 4K and Supplemental Figure 4D). While this ICC is relatively robust, this analysis indicates that over 10% of the variance in the SI can be attributed to RNA splicing changes in the skeletal muscle occurring during the 3M interval. Moreover, within the cohort we found that individuals with SI scores higher than 0.8 displayed minimal longitudinal variation (Figure 4, I and K). As these individuals already possessed severely dysregulated splicing at the BL assessment, there is limited capacity for further shifts in Ψ in response to changes in free [MBNL] and the overall score to increase. Exclusion of these severely affected individuals substantially reduced between-biopsy agreement (ICC = 0.703, *P* < 0.0001) (Supplemental Figure 8), indicating general asymmetric variation of splicing dynamics over 3M across the spectrum of disease severity.

### The SI stratifies DM1 individuals across the spectrum of disease severity.

Given the mean increase in SI observed over 3M, we sought to define subgroups of DM1 individuals according to their BL SI to facilitate evaluation of splicing dynamics over this time course. Rather than rely on visual inspection of the SI distribution to define potential subcohorts, we applied 2 related but distinct data-driven approaches to identify empirical phenotype clusters that could then be evaluated for clinical validity. First, we conducted a latent class analysis (LCA) using Ψ values from 5 SI events (*CAMK2B*, *CCPG1*, *DMD*, *INSR*, and *GOLGA4*) that displayed modest correlations with the composite measure. The LCA was run using either the entire cohort with available targeted RNA-seq data or those DM1 participants with matched BL outcome measures (sensitivity analysis, *n* = 52). Overall, the fit indices suggested a 3-class solution that loosely corresponded to low, moderate, and high global SI values. The class compositions did not significantly differ with respect to biological sex (*P* = 0.21) or age (*P* = 0.24), but statistically significant differences in concurrent measures of function were observed (3-class solution, BL only) (Supplemental Figure 9 and Supplemental Table 8). Secondary application of *k*-means fold clustering identified similar bounds of SI scores as in the LCA 3-class solution (Group 1 SI mean = 0.21, span = 0.01–0.39; Group 2 SI mean = 0.60, span = 0.41–0.75; Group 3 SI mean = 0.92, span = 0.77–1.0). In combination, these analyses allowed for definition of 3 SI-stratified subcohorts: SI_Mild_ = 0–0.4, SI_Moderate_ = 0.41–0.75, and SI_Severe_ = 0.76–1.0.

**Figure 5:**
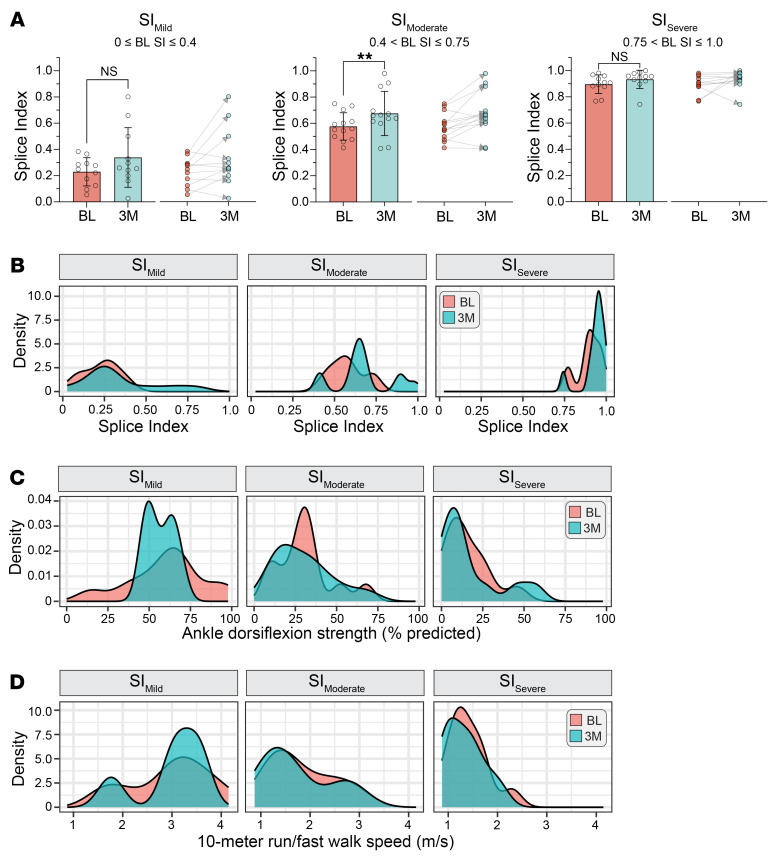
Splice Index (SI) demonstrates dynamic shifts over 3 months in select DM1 subcohorts stratified by baseline SI score. (**A**) SI score significantly increases between baseline (BL) and 3 months (3M) in SI_Moderate_ group. DM1 participants in longitudinal cohort were stratified by BL SI score: SI_Mild_ (0 ≤ BL SI ≤ 0.4, *n* = 11), SI_Moderate_ (0.4 < BL SI ≤ 0.75, *n* = 13), and SI_Severe_ (0.75 < BL SI ≤ 1.0, *n* = 11). Data represented as mean ± SD. **P < 0.01 by paired, 2-tailed *t* test. NS, not significant. Connected scatter plots are also displayed to show shifts in BL and 3M paired SI values for each DM1 individual. (**B**) Kernel density estimation plot of BL and 3M SI distributions in SI_Mild_, SI_Moderate_, and SI_Severe_ groups. (**C** and **D**) Kernel density estimation plot of BL and 3M (**C**) ADF and (**D**) 10MRW distributions in SI_Mild_, SI_Moderate_, and SI_Severe_ groups. ADF is reported as the percentage of predicted strength as compared with unaffected individuals and 10MRW in speed (m/s).

All 35 DM1 participants within the longitudinal subcohort (Figure 1A) were evenly distributed among the SI_Mild_ (*n* = 11), SI_Moderate_ (*n* = 13), and SI_Severe_ (*n* = 11) subgroups according to their BL SI. Consistent with our cross-sectional analyses, no significant differences in mean outcome measures were observed between time points across all 3 groups except for a modest change in HGS for SI_Moderate_ and KE for SI_Mild_ (Supplemental Figure 10). However, SI_Moderate_ individuals exhibited a significant increase in SI scores at 3M (mean ΔSI = 0.1) (Figure 5A). Although the SI_Mild_ group also possessed a mean increase in SI, this change was not statistically significant, likely due to the wide distributional shift of SI values as visualized by kernel density plot. As expected, participants classified as SI_Severe_ showed minimal changes in SI scores (Figure 5, A and B). Overall, these findings suggest that the SI can sensitively detect longitudinal changes in RNA mis-splicing within participants, and that these changes appear to be more pronounced in individuals with moderate spliceopathy.

### Individual RNA splicing events facilitate detection of temporal mis-splicing dynamics in SI-stratified groups.

**Figure 6:**
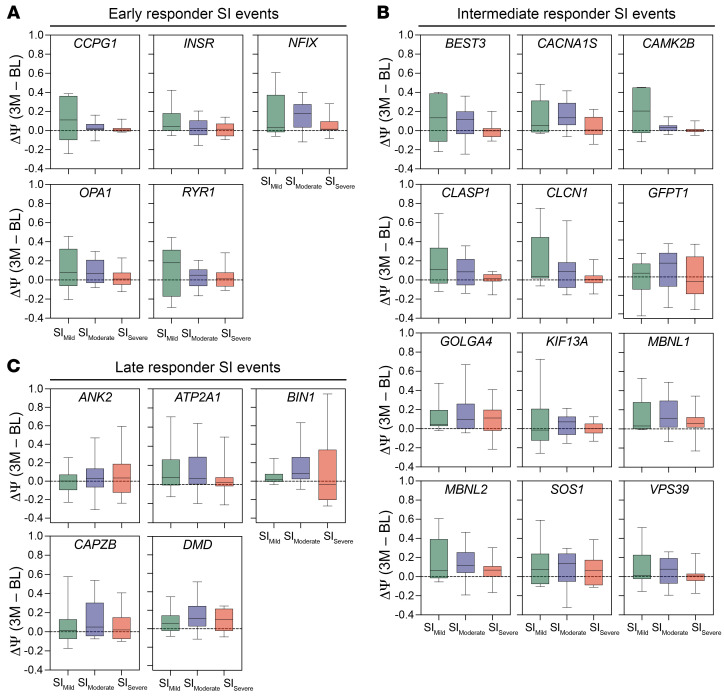
Variable sensitivity of 22 events within Splice Index (SI) panel capture RNA splicing shifts over 3 months in baseline SI–stratified subcohorts. (**A**–**C**) Relative sensitivity of RNA splicing events included in SI panel to levels of free [MBNL] allows for detection of changes in mis-splicing across the spectrum of DM1 splicing dysregulation. Normalized ΔΨ (3M – BL) derived from targeted RNA-seq (Supplemental Table 5) of (**A**) early, (**B**) intermediate, and (**C**) late responder RNA splicing events as defined in Figure 2. Data presented as box-and-whisker plots, where lines represent median ΔΨ, the bounds of the boxes are the 25th and 75th percentiles, and whiskers extend to maximum and minimum values. ΔΨ for SI_Mild_, SI_Moderate_, and SI_Severe_ subcohorts are displayed for each event.

Given the dynamic changes in the SI observed in DM1 individuals over 3M, we aimed to evaluate the sensitivity of early, intermediate, and late responsive splicing events encompassed within the SI panel to assess longitudinal Ψ shifts in SI-stratified Mild, Moderate, and Severe subcohorts, respectively. Normalized ΔΨ (3M – BL) for all 22 events is displayed using box-and-whisker plots within these subcohorts (Figure 6). Overall, event response classifications accurately reflected the sensitivity of these splicing events to detect Ψ changes in the corresponding range of the disease spectrum. Early responder events (*INSR* and *CCPG1*) showed the broadest distribution of changes in SI_Mild_, while late responder splicing events (*BIN1* and *ANK2*) sensitively detected Ψ changes at 3M in the most severely impacted DM1 participants (SI_Severe_). Intermediate events detected subtle change across all SI subcohorts, with variable effect.

In concordance with the overall increase in the SI over 3M in the full longitudinal cohort, normalized ΔΨ generally increased across all events within each BL SI–stratified group. However, reversion of mis-splicing was also observed, particularly in SI_Mild_; this observation aligns with the mixed trajectory of SI scores within individuals in this group (Figure 5A, connected scatter plot). Consistent with maximal splicing dysregulation being reached for most events in the SI_Severe_ subgroup, the widest distributions of Ψ changes between time points were observed within late responder events responsive to low levels of free [MBNL]. These observations demonstrate how variably sensitive individual splicing events within the composite biomarker may respond to therapeutic target engagement across the spectrum of DM1 spliceopathy using natural RNA mis-splicing progression as a proxy.

### Combination of BL SI and outcome measures may be predictive of 3M performance.

Stratification of the longitudinally sampled DM1 cohort by BL SI revealed that the significant increase in this biomarker over 3M was primarily driven by those individuals with moderate splicing dysregulation (SI_Moderate_). While almost no significant differences in mean outcome measures were observed within the 3 subcohorts (Supplemental Figure 10), an examination of the distribution of performance measures from participants within each SI-stratified subcohort at both BL and 3M revealed a lack of comparable stratification in the performance range of individuals. This observation was particularly evident for ADF and 10MRW, where SI_Mild_ and SI_Moderate_ subcohorts encompassed participants with varying measures of muscle strength and motor function spanning the full spectrum of disease severity (Figure 5, C and D). In contrast, a narrow distribution of outcome measure performance centered at the most severe end of the disease spectrum was observed for the SI_Severe_ group, consistent with the stability of the SI over 3M (Figure 5, C and D). This is consistent with these individuals reaching the upper limit of measurable deficits in muscle strength and motor function using these assessments.

These analyses suggest that due to the heterogeneity of disease presentation, especially for less severely affected individuals, splicing dysregulation as measured by SI may be less representative of phenotypic performance in our stratified subcohorts at the time of biopsy collection than previously indicated by the full cross-sectional analysis (Figure 3). To evaluate this further, we conducted a time point comparative analysis of SI scores and performances measures in the longitudinal cohort. BL SI scores were compared to time point–matched and 3M outcome measures, and vice versa, to assess the temporal relationship between splicing dysregulation and functional changes. BL SI scores exhibited stronger associations for nearly all 3M outcomes – ADF, 10MRW, KE, and vHOT_Middle Finger_ – compared with time point–matched measures. Conversely, correlations weakened when assessing associations between 3M SI scores and BL outcomes (Supplemental Figure 11). While the sample size for this analysis was small and changes in relative associations were modest, these results collectively suggest that changes in splicing dysregulation as reflected by the SI may precede detectable phenotypic changes. This is perhaps due to a time delay between aberrant exon inclusion patterns and downstream biological deficits that influence functional performance.

**Figure 7:**
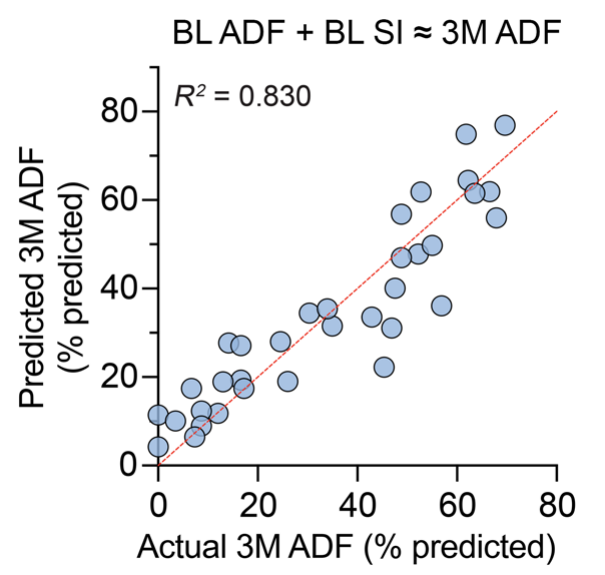
Multiple linear regression modeling highlights prognostic utility of the Splice Index (SI) in combination with time point–matched outcomes to predict future function at 3 months. Combination of baseline (BL) SI and time point–matched measure of ankle dorsiflexion (ADF) strength is predictive of performance at 3 months (3M). Agreement plot of actual versus predicted 3M ADF derived from multiple linear regression model with adjusted *R*^2^ are displayed (Supplemental Figure 12, Model 1). All quantitative parameters are provided in Supplemental Table 9.

Given the results of our time point correlative analysis, we assessed the prognostic utility of the SI for predicting short-term functional outcomes in DM1 individuals in the absence of therapeutic intervention. We chose to use ADF as the primary outcome given its close relationship with strength of the TA, the muscle biopsied for derivation of SI. Multiple linear regression modeling demonstrated that while BL ADF alone accounts for a significant portion of the variance in 3M ADF performance (Model 2: BL ADF ≈ 3M ADF), adjusted *R*^2^ = 0.797), inclusion of BL SI as a covariate significantly increased the power of the model (Model 1: BL ADF + BL SI ≈ 3M ADF, adjusted *R*^2^ = 0.830) (Figure 7 and Supplemental Figure 12). While this change was subtle, these analyses highlight the predictive power of the SI when combined with measures of muscle strength to predict future outcomes in DM1 individuals.

## Discussion

These results describe the use and validation of a robust, reliable, and sensitive DM1 composite RNA splicing biomarker – the Myotonic Dystrophy Splice Index. The DM1 SI accurately distills global RNA mis-splicing severity in skeletal muscle across the disease spectrum and has concurrent validity with measures of muscle strength and function. Additionally, early analyses indicate the DM1 SI may have prognostic utility for prediction of future function.

RNA splicing is well recognized as a core pathogenic mechanism in DM1 (11). Prior preclinical studies have demonstrated that RNA splicing events are differentially sensitive to the degree of MBNL1 sequestration (22, 31). This molecular variability has posed a challenge in developing a single splicing event–based biomarker for use in analyzing therapeutic efficacy (30, 43). DM1 phenotypic heterogeneity has further complicated clinical trial design by inflating study enrollment and limiting the ability to detect dose-response relationships and associations between changes in RNA splicing and clinical performance. To develop meaningful endpoints in a heterogeneous disease population, a biomarker should be able to account for variance at entry and variability in rates of disease progression. These barriers have been overcome by the development of an aggregate measure of RNA splicing inclusive of splicing events with variable responsiveness across the disease spectrum. Work described here has demonstrated that the SI is a durable measure of disease-specific splicing dysregulation and is strongly associated with multiple measures of physical function (Figures 1–3).

The SI demonstrates high technical replication and reproducibility supported through high-resolution coverage of splice isoform–specific transcripts using targeted RNA-seq and subsequent use of a standardized set of normative reference Ψ values. These reference values provide the basis for even scaling of each event’s Ψ across the complete spectrum of splicing dysregulation observed in DM1 and equal weighting in the final, averaged metric of 22 events. This is divergent from methodologies previously used to capture DM1 mis-splicing such as [MBNL]_inferred_, which require a well-balanced set of internal DM1 standards for Bayesian inference modeling to accurately define an individual’s level of RNA mis-splicing (22). As this methodology is sensitive to appropriate sampling of DM1 individuals across the spectrum of disease for appropriate scaling, changes due to therapeutic engagement may be inappropriately exaggerated or lost, especially when using a small sample size. Development of standardized, normative reference Ψ values from a large, well-phenotyped DM1 cohort like that described here overcomes this barrier in biomarker use, allowing for consistent, reliable evaluation and scaling of an individual’s Ψ level of RNA mis-splicing.

Adults with DM1 report issues associated with mobility, ambulation, or hand function consistently among the top 5 most impactful symptoms (44, 45). Correspondingly, the SI has concurrent validity with measures of muscle strength and ambulation, as demonstrated by strong associations with HGS, ADF strength, and the 10MRW (Figure 3). The SI also correlates modestly with myotonia, as assessed by vHOT (Supplemental Figure 5). Therapeutic administration in DM1 preclinical models indicates that myotonia is a rapidly reversible phenotype, supporting the idea that myotonia outcome measures may provide a rapid assessment of therapeutic target engagement in human clinical trials (25, 27, 29, 46). The diminished strength of association with the SI is likely related to the challenge of quantifying this phenotype in a reliable fashion rather than lack of relevance to the underlying pathogenic mechanism of disease, especially as phenotype-causative events are included within the SI splicing event panel (*CLCN1* and *CACNA1S*) (16–18, 20, 21). Future efforts aimed at improving reliability of this outcome measure are needed to fully assess correlation with the SI. Additionally, as the SI is designed to capture changes associated with skeletal muscle performance, we would expect to observe limited associations with other measures of multisystemic dysfunction, such as cognition. While the same methodologies may be applied to develop composite splicing indices indicative of disease severity in other organ systems, the limited accessibility or feasibility in accessing target biospecimens, including samples from the GI tract and CNS, may limit the development of parallel biomarkers.

One key observation from these analyses is that RNA splicing in DM1 skeletal muscle is more dynamic over short time periods than previously anticipated, particularly among individuals with moderate SI scores. The clinical progression of DM1 is measured over years and it is unexpected that significant differences in performance would be observed over 3M (40–42). This was borne out by clinical observations in the assembled study cohort (Figure 4 and Supplemental Figure 6). However, assessment of longitudinal variation in the SI indicates that RNA mis-splicing may fluctuate over time. Importantly, these splicing shifts portend a significant decline in quantifiable measurable performance, specifically for those individuals with mild and moderate spliceopathy (SI_Mild_ and SI_Moderate_) (Figure 5A and Figure 6). These findings have important implications for clinical trial design, specifically in regards to enrollment size required to detect significant therapeutic efficacy above natural shifts in RNA splicing. This is especially important, as SI_Mild_ and SI_Moderate_ classified individuals may be the most responsive to disease-modifying interventions as measured by RNA splicing events included in the SI. However, these participants also have a heterogeneous distribution of individual performance on outcome assessments (Figure 5 and Supplemental Figure 10). Future studies in an expanded DM1 cohort are needed to determine how to best identify these individuals with a range of responsive spliceopathy amenable to therapeutic-mediated modulation via clinical outcome assessments alone. Early analyses indicate that inclusion of early, intermediate, and late responder RNA splicing events within the composite SI show promise in detecting splicing changes across the complete molecular and phenotypic distribution of DM1 disease presentation (Figure 6). These splicing events may have utility when evaluating therapeutic efficacy in affected individuals with variable disease severity in post hoc analyses (29, 47). Despite the observed dynamics of RNA mis-splicing in our longitudinal cohort, extended longitudinal studies with increased participant numbers are needed to fully evaluate and describe temporal fluctuations of RNA mis-splicing and the stability of the SI.

Time point correlation analysis suggested that splicing dysregulation as measured by the SI may precede detectable phenotypic changes. BL SI scores exhibited subtle, but stronger associations with 3M functional outcomes compared with time point–matched measures (Supplemental Figure 11). One hypothesis for this difference is that the RNA transcripts measured in this assay are not yet translated to protein, and therefore represent a future composition of muscle function. The time from transcription to translation of the RNA targets may occur over a variable period, but are subsequently accounted for in the strength and functional measures at 3M. This suggests that the SI in combination with BL functional measures may be predictive of future functional performance, and our regression analyses support this prognostic utility (Figure 7 and Supplemental Table 9). The ability to predict long-term outcomes (e.g., >12 months) is an open question that requires additional, longer-term studies.

While analyses here validate the utility of the SI for adult-onset DM1, we have preliminarily evaluated the potential validity of the SI to, at minimum, capture disease-specific splicing dysregulation in a small cohort of congenital myotonic dystrophy (CDM) and ambulatory DM2 participants (*n* = 8 CDM and 4 DM2). The Ψ values of the 22 events encompassed within the SI effectively cluster these DM subtypes along the range of splicing dysregulation observed in our DM1 cohort. This analysis also provided an initial indication that the SI can sensitively assess temporal changes in global mis-splicing in these populations; 2 CDM children with longitudinal biopsies displayed agreement with previously described shifts in global splicing dysregulation during pediatric development (Supplemental Figure 13) (31). Although genotyped and aged-matched normative reference Ψ values would be required for use of the SI in these DM subtypes, these data show promise that the splicing events used to generate the DM1 SI or a modified event panel could be developed for these populations. An expanded cohort of well-phenotyped CDM and DM2 participants would be required to evaluate the correlation of this biomarker to muscle performance using well-defined clinical outcome assessments for these participants.

In summary, the SI is a robust, reliable, and sensitive biomarker that captures dynamic RNA splicing changes in DM1 skeletal muscle, is associated with physical strength and ambulation, and is potentially predictive of future function. These properties position the SI as a valuable biomarker for assessing disease severity and therapeutic target engagement in clinical trials. Future studies should focus on validating the SI in a secondary DM1 cohort, evaluating the biomarker’s stability over extended periods of disease progression, and assessing its application across other DM subtypes.

## Methods

### Sex as a biological variable.

Our study utilized muscle biopsies from a relatively even distribution of both adult male and female individuals with DM1 and unaffected/disease control participants. Analysis of key variables presented here did not find differences regarding sex.

### Participants and muscle biopsy collection.

Participants with clinically or genetically diagnosed DM1 over the age of 18 were enrolled in END-DM1 (ClinicalTrials.gov NCT03981575) or a prior pilot study (HELP-DM1). Individuals were required to be ambulatory and have sufficient TA muscle bulk for muscle sampling. To participate in the muscle biopsy within the END-DM1 study, all participants’ ADF strength had to be rated between 4+ and 4– on the Medical Research Council (MRC) scale of muscle strength by the study investigator. Muscle biopsies were not performed in individuals with a history of bleeding disorders, a history of anticoagulation or antimyotonia medication use, or a platelet count less than 50,000.

Muscle biopsies from DM1 participants were collected with either a 14-gauge Argon Supercore needle or a Bergstrom needle of the TA by previously described techniques (29). At the 3M longitudinal assessment, select biopsies were conducted on the ipsilateral or contralateral TA. Select unaffected AdCo and CDM biopsies of the TA, vastus lateralis, and soleus were collected as previously described (31). Additional AdCo, DM2, DMD, and LGMD biopsies were collected from the TA of ambulatory participants with a 14-guage needle as part of an in-house biorepository study (CIMR Neuromuscular Research Biobank). Available demographic information of all available participants is listed in Supplemental Table 1.

### Functional outcome measure assessment and analysis.

QMT of HGS, ADF, and KE strength were conducted with a fixed quantitative system (29). Strength measures are reported as the average percentage of predicted strength of both the left and right limb as compared with non-affected individuals of the same age, sex, and height (29). TMTs included the 10MRW, which were conducted as previously described and times were used to calculate fast walk/running speed (meters/second) (48). Myotonia was assessed using the vHOT assessment (29). In brief, participants are instructed after resting their dominant hand in an open position for 5 minutes on a flat surface to make a fist and squeeze for 3 seconds. Participants are then cued to open their hand completely until all fingers and thumb are fully extended and relaxed. A blinded rater reviewed video of the assessment and measured the time from when the participant is cued to open the hand until the middle finger is fully extended (vHOT_Middle Finger_) and until the thumb is fully extended (vHOT_Thumb_). Following a 5-minute rest, a second trial was completed. Times expressed in seconds from the second trial for both vHOT_Middle Finger_ and vHOT_Thumb_ were used for analyses. All measures are reported in Supplemental Table 1.

### Muscle biopsy RNA extraction and quality control.

RNA was extracted from biopsies via a TRIzol/chloroform extraction supplemented with bead-based tissue homogenization (Benchmark Scientific) and purified using the Zymo Clean and Concentrator – 5 kit following the standard protocol for on-column DNase treatment. RNA quality and abundance were assessed using fragment analysis (Agilent Fragment Analyzer 5200, DNF-472 [15 nt] RNA kit) and Qubit 4 Fluorometer (Invitrogen). Most RNA samples had RIN values greater than 7, but samples were utilized for subsequent analyses if they had RIN scores greater than 5.5. RNA was stored at –80°C until further processing.

### Total RNA-seq library preparation, sequencing, and RNA splicing analysis.

Libraries were prepared using 100–250 ng of RNA with the NEBNext rRNA Depletion Kit in conjunction with the NEBNext Ultra II Directional RNA Library Prep Kit for Illumina (New England Biolabs). Deviations from the manufacturer’s protocol include modifications of the recommended 5-fold adaptor dilution to a 40-fold adaptor dilution. Libraries were paired-end (2 × 76 bp) sequenced on the Illumina NextSeq 2000 using P3 Reagents at a loading concentration of 750 pM with a 1% PhiX spike-in. Samples were sequenced for coverage at more than 65 million reads. Differential splicing analysis for total RNA-seq data was performed using rMATS, as previously described (31). Event Ψ was calculated and deemed significantly mis-spliced when |ΔΨ| was 0.1 or greater and FDR was 0.05 or less between defined groups. Estimated levels of free [MBNL] ([MBNL]_inferred_) were derived as previously described using all DM1 and AdCo samples (*n* = 117) (22, 31). For splicing event classification, Ψ values derived from total RNA-seq (Supplemental Table 3) were plotted against the [MBNL]_inferred_ and fit to a 4-parameter dose-response curve as previously described to derive quantitative parameters of splicing shifts over the disease spectrum (Supplemental Table 4) (22, 23).

### Targeted RNA-seq library preparation and sequencing.

A targeted RNA-seq library was designed similarly to that previously described for generation of a species-specific SI in DM1 mouse models (28). In brief, RNA splicing events identified using the total RNA-seq dataset as having a large, DM1-specific effect size, low Ψ variability in AdCo participants, and a gene structure amenable to amplicon sequencing were included in the final 22-event panel (29). Specific splicing events that were previously identified as mis-spliced in DM1 patients and were moderately correlated with manual muscle testing of the ADF in DM1 participants were also included despite smaller effect sizes (30).

cDNA was synthesized from 10 ng of high-quality RNA using the SMART-Seq v4 Ultra Low Input RNA Kit (Takara). Genome coordinates for alternative exons and PCR primers for amplifying cDNA are listed in Supplemental Table 11. To normalize a balance of sequence reads across the 22 splicing events, especially for low-abundance transcripts, the 22 primer sets were distributed across 2 multiplex, first-stage PCR reactions of 14 and 16 cycles (PCR1 A and B, respectively). All PCR1 primers included a 5′ adapter sequence for incorporation of sample barcodes and priming sites for Illumina sequencing in a second-stage PCR (PCR2). Libraries were individually normalized based on concentrations assessed using the Qubit 4 Fluorometer (Invitrogen) and average library size as determined by smear analysis (Agilent Fragment Analyzer 5200, DNF-474 HS NGS kit). Libraries were paired-end (2 × 151) sequenced on the iSeq 100 using an i1 Reagent v2 (300-cycle) kit at a loading concentration of 125 pM with a 10% PhiX spike-in.

### Quantification of SI.

Amplicon library reads in raw FASTQ format were aligned to a custom reference sequence set of exon inclusion and exclusion isoforms (Supplemental Table 11) using HISAT2 v2.1.0 (http://daehwankimlab.github.io/hisat2/) and filtered reads with the -no-spliced-alignment flags and -no-softclip run parameters. Primary aligned reads spanning splice junctions were subset from the resulting bam files using a combination of Samtools v1.7 (https://github.com/samtools/) and Bash (shell program) command line interface (CLI). Isoform-specific reads with unambiguous alignment were counted and further processed in a custom Python script to derive Ψ for each splicing event. Ψ values were calculated as the fraction of exon inclusion isoform reads relative to all exclusion and inclusion isoform counts. The SI score was then calculated as follows. For each participant *y*, normalized Ψ values were calculated for each splicing event *z* as (Ψ_*y*, *z*_ – Ψ_Median Control, *z*_)/(Ψ_DM95, *z*_ – Ψ_Median Control, *z*_) where Ψ_Median Control, *z*_ is the median Ψ for event *z* in unaffected healthy adults, and Ψ_DM95, *z*_ is the 95th percentile for most severely affected DM1 individuals (28). Normative values used throughout these analyses unless otherwise annotated were derived from our cohort of AdCo samples (*n* = 22) and all DM1 individuals subjected to targeted RNA-seq (*n* = 95) (Supplemental Table 6). Normalized Ψ values for each participant *y* were then averaged to generate a linear value from 0 to 1. SI values slightly below zero or above 1 due to variable RNA splicing relative to normative reference were rounded to the nearest integer.

### Statistics.

Clinical data were extracted from Redcap using Microsoft Excel version 16.75. Any clinical data points that were available but deemed to be questionable by the study evaluators were subsequently reviewed by the study’s Principal Investigator (PI). An ultimate decision regarding inclusion/exclusion was made jointly by the evaluators and PI for these specific data points. Significant differences were determined by paired *t* test or 1-way ANOVA with Tukey’s correction, as described in figure legends using GraphPad Prism 10.1.0. Univariate correlations and multiple linear regression modeling were performed using GraphPad Prism and R statistical software (R 4.2.3). Univariate Pearson’s or Spearman’s correlation coefficients were calculated depending on the distributional properties of the variable, and significance is reported with a 2-tailed *P* value and 95% confidence interval (CI). Multiple linear regression modeling assumed a least squares regression and the adjusted *R*^2^ was used to quantify goodness of fit. Assumptions of regression, including normality of residuals, were assessed for each model. Intraclass correlation coefficients were calculated using a 2-way fixed-effects model for absolute agreement between measures. *P* values of less than 0.05 were considered statistically significant for all analyses.

LCA was performed in the R statistical environment (v4.2.1) using poLCA (49). First, the correlation structure of all 22 RNA-seq Ψ values was examined to quantify the extent of collinearity. This preanalysis step is standard for LCA because the approach is sensitive to multicollinearity. Based on the correlations, 5 SI splicing events exhibiting only modest correlations were selected (*CAMK2B*, *CCPG1*, *DMD*, *INSR*, and *GOLGA4*). Next, the mean SI value for each event was calculated using the entire cohort. For each individual, their continuous SI value for each event was dichotomized such that 1 indicated a value greater than the mean SI for that event and zero indicated less than the mean value. The LCA was run using both the entire cohort with available targeted RNA-seq data (*n* = 129) and those adult-onset-DM1 individuals with matched BL measures only (*n* = 52) (sensitivity analysis). We chose “any class size smaller than 10% of the total sample size” as a predetermined stopping point as the threshold to stop testing additional classes. Based on this stopping criterion, 2-, 3-, and 4-class models were tested, but only 2- and 3-class solutions were interpreted. Each model was run 300 times, with 5,000 set as the maximum number of iterations of estimation algorithm cycles to increase the odds of finding the best global maximum of the log-likelihood function. Goodness of fit was assessed using Akaike information criteria and Bayesian information criteria. Predicted class membership was validated using concurrent and future functional measures of strength and mobility to evaluate the potential clinical utility of these data-driven phenotypes. Bounds of 3 defined SI subcohorts were determined using sklearn’s KMeans clustering package (https://scikit-learn.org/stable/modules/generated/sklearn.cluster.KMeans.html). The supervised model grouped samples by SI score into Mild, Moderate, and Severe categories. Minimum and maximum scores in each class informed the boundaries of their respective groups.

### Study approval.

All studies were approved by the IRB at the University of Utah (HELP-DM1, IRB_00079580) or WIRB Copernicus Group IRB (END-DM1, IRB_20204399 and CIMR Neuromuscular Research Biobank, IRB_20204038). Written informed consent was obtained from all participants prior to any study procedures or biospecimen collection.

### Data availability.

All data points presented in the graphs are detailed in the supplemental material and Supporting Data Values file. RNA-seq datasets and computer code scripts used for analysis in this study can be found in the following databases and repositories. Total and targeted RNA-seq libraries: NCBI SRA PRJNA1079722 and PRJNA830511 (https://www.ncbi.nlm.nih.gov/bioproject/PRJNA1079722 and https://www.ncbi.nlm.nih.gov/bioproject/?term=PRJNA830511). Analysis scripts: https://github.com/Center-for-Inherited-Muscle-Research/splice_index

## Author contributions

MP, CAT, NEJ, and MAH conceptualized the study. NEJ collected all biospecimens and served as the clinical study principal investigator. MP, AG, and MAH processed biospecimens and performed RNA-seq experiments. AJ, AB, KNB, JD, and MK collected and scored clinical data. MP, KI, KB, JMH, and MAH curated clinical data for analyses. KI and KB performed bioinformatic analyses. MP, KI, MH, DML, and MAH performed data analyses. CAT and NEJ acquired funding. NEJ and MAH were project administrators and supervised the study. MAH, MP, KI, and DML wrote the original draft of manuscript and generated all figures. All authors reviewed and edited the manuscript.

## Supplementary Material

Supplemental data

ICMJE disclosure forms

Supplemental table 1

Supplemental table 2

Supplemental table 3

Supplemental table 4

Supplemental table 5

Supplemental table 6

Supplemental table 7

Supplemental table 8

Supplemental table 9

Supplemental table 10

Supplemental table 11

Supporting data values

## Version 1 01/21/2025

In-Press Preview

## Version 2 02/17/2025

Electronic publication
