# Supplementary material for: The Splice Index as a prognostic biomarker of strength and function in myotonic dystrophy type 1
Source: J Clin Invest. 2025 Jan 7;135(4):e185426. doi: 10.1172/JCI185426 (PMC11827844; doi:10.1172/JCI185426)
Supplement: Supplemental data [file jci-135-185426-s174.pdf]

Supplemental Figure 1

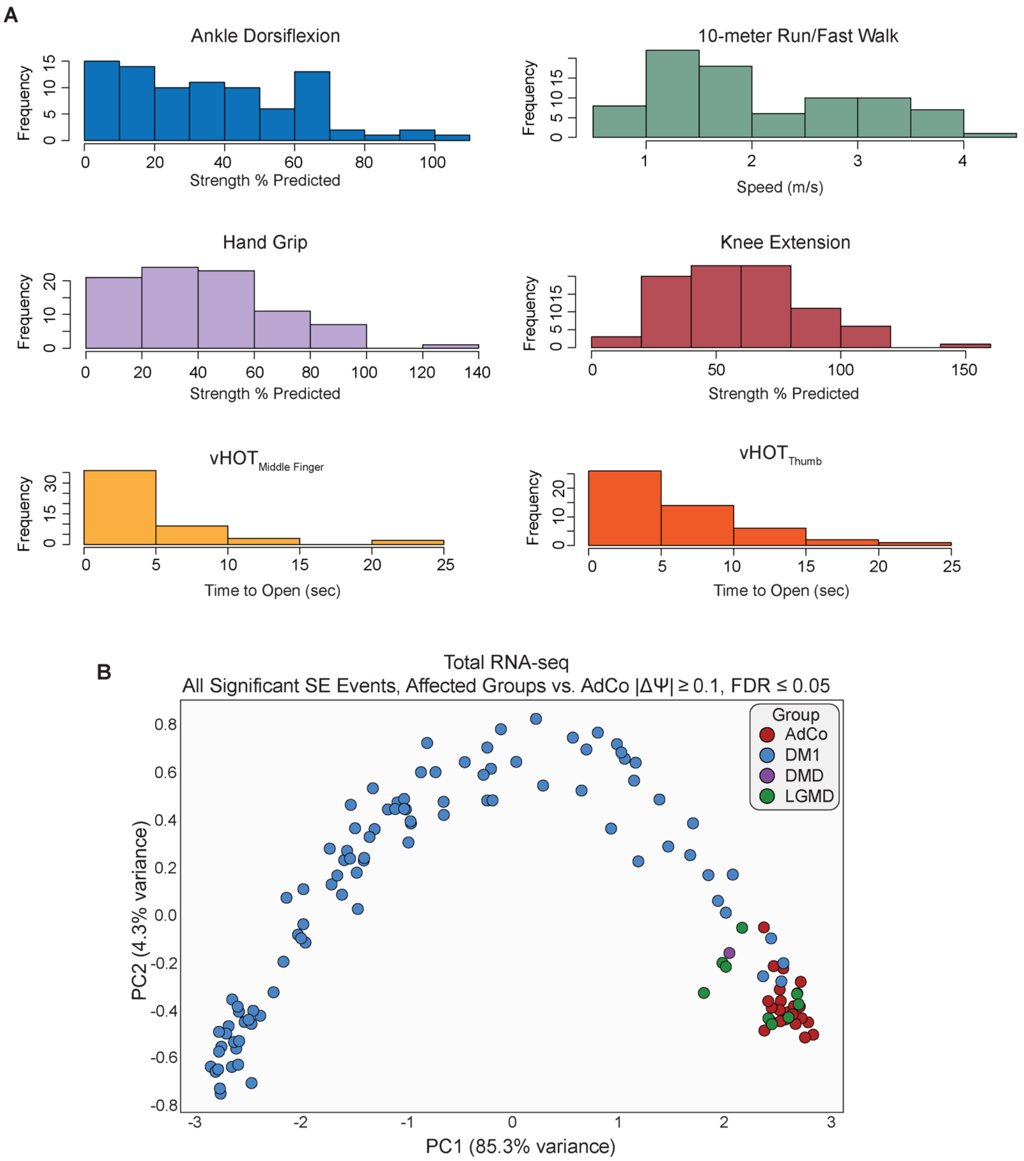

Supplemental Figure 1. Phenotypic and molecular heterogeneity of DM1 participants subjected to total RNA-seq. (**A**) Distribution of performance measures captured in all DM1 participants via quantitative muscle testing (ankle dorsiflexion, hand grip strength, knee extension), timed motor test (10-meter run/fast walk), and myotonia (video hand opening time – vHOT). Measures are reported as the percent predicted strength as compared to unaffected individuals, speed in m/s, and time to open closed fist (seconds), respectively. (**B**) Principal component analysis of all significantly dysregulated skipped exon (SE) events in muscle biopsies subjected to total RNA-seq between DM1 versus unaffected adult controls (AdCo) and disease control reference groups (DMD & LGMD). In total 946 significantly mis-spliced, skipped exon events were defined by $|\Delta\Psi| \geq 0.1$, FDR ≤ 0.05 and are listed in Supplemental Table 2.

Supplemental Figure 2

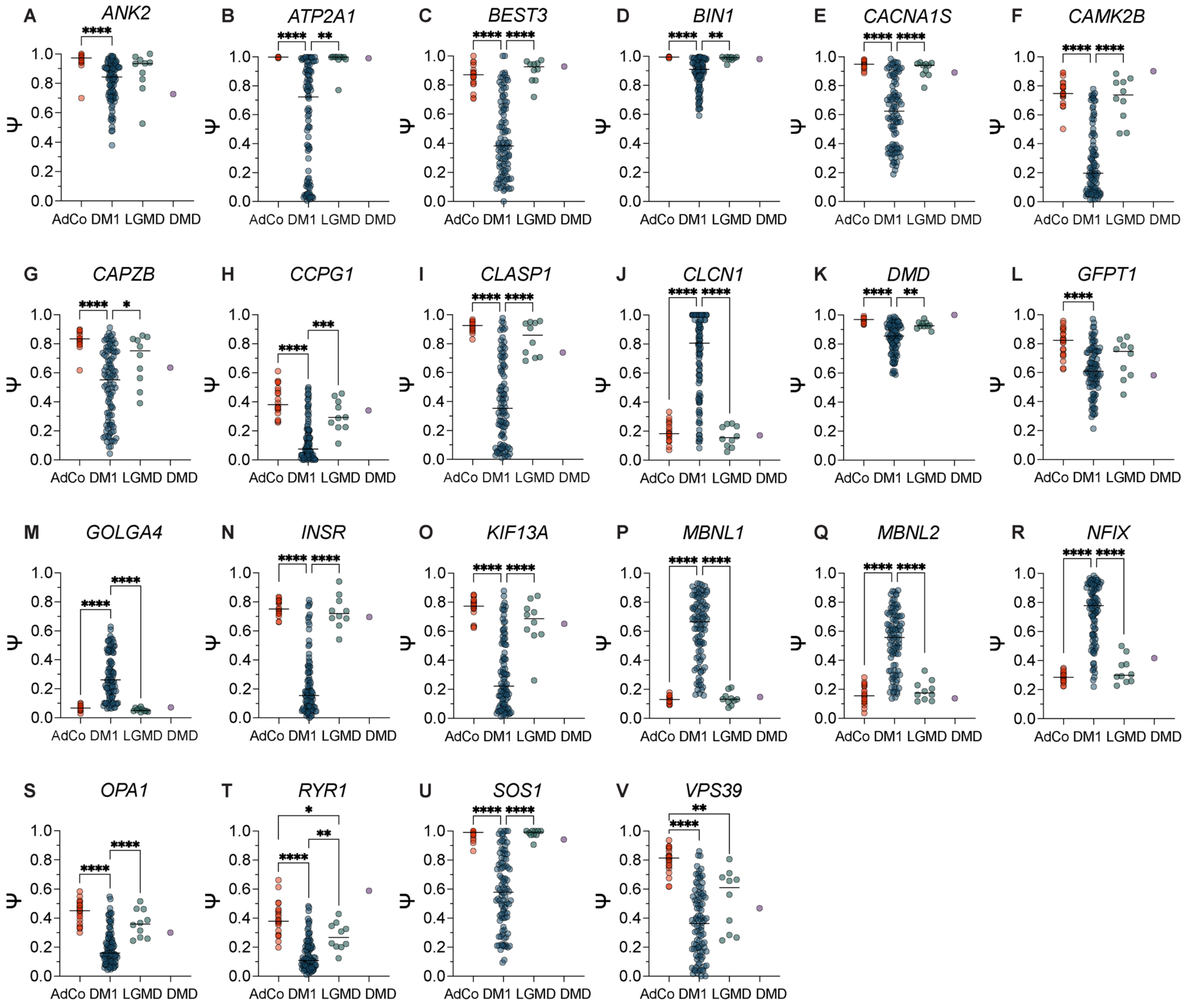

Supplemental Figure 2. 22 splice events selected for composite Splicing Index are disease-specific and display minimal variability in unaffected adult controls and disease controls. (**A – V**) Strip plots of Ψ values from total RNA-seq for 22 skipped exon events in composite Splicing Index panel. Ψ values (Supplemental Table 3) are colored by sample group (n = 22 AdCo, n = 95 DM1, n = 10 LGMD, and n = 1 DMD). Bar represent median Ψ; **** $p < 0.0001$, *** $p < 0.001$, ** $p < 0.01$, * $p < 0.05$, One-way ANOVA with Tukey's correction where DMD was not included in analysis due to insufficient sample size. All comparisons not shown between AdCo and LGMD were not significant.

Supplemental Figure 3

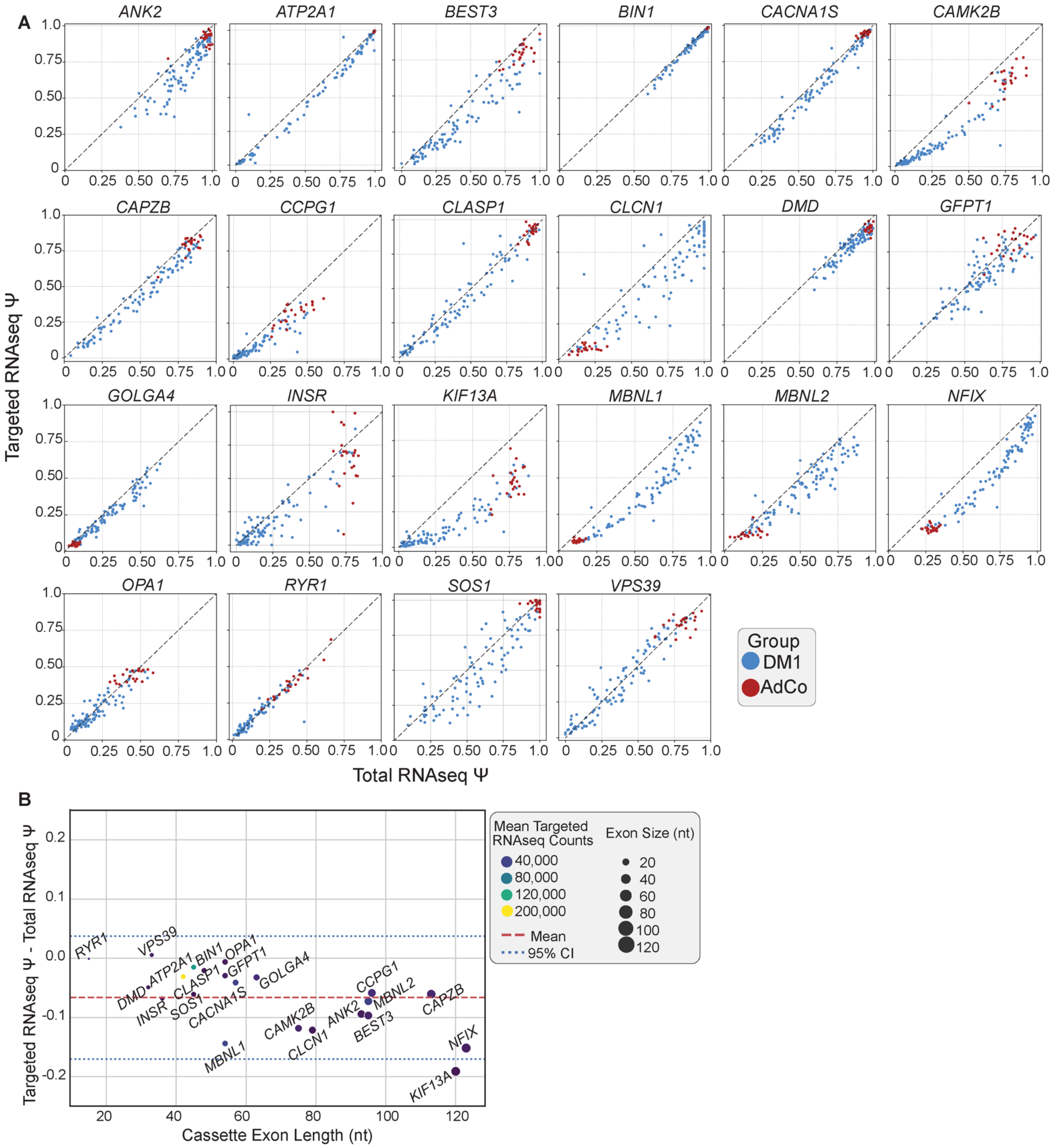

Supplemental Figure 3. Targeted RNA-seq shows limited under-detection bias of exon inclusion isoforms for select splice events included within the Splicing Index. (**A**) Comparison of Ψ estimates derived from total and targeted RNA-seq for panel of 22 skipped exon RNA splicing events included in Splice Index panel. Ψ values are reported in Supplemental Table 3 & Supplemental Table 5. Line of agreement (x = y) is displayed to show relative detection bias of smaller, exon exclusion isoforms in targeted RNA-seq and subsequent lower Ψ compared to total RNA-seq; n = 95 DM1 & 22 AdCo. (**B**) Targeted RNA-seq shows small bias for exon exclusion isoforms that is moderately correlated to cassette exon length. Red dotted line represents mean Ψ difference between methodologies and blue dotted lines represent the mean ± one standard deviation. Size of each event dot is reflective of exon length and color is reflective of mean event counts in targeted RNA-seq. Mean difference between targeted and total RNA-seq for each splice event was derived from all DM1 and AdCo samples subjected to both sequencing methodologies (n = 95 DM1 & n = 22 AdCo).

Supplemental Figure 4

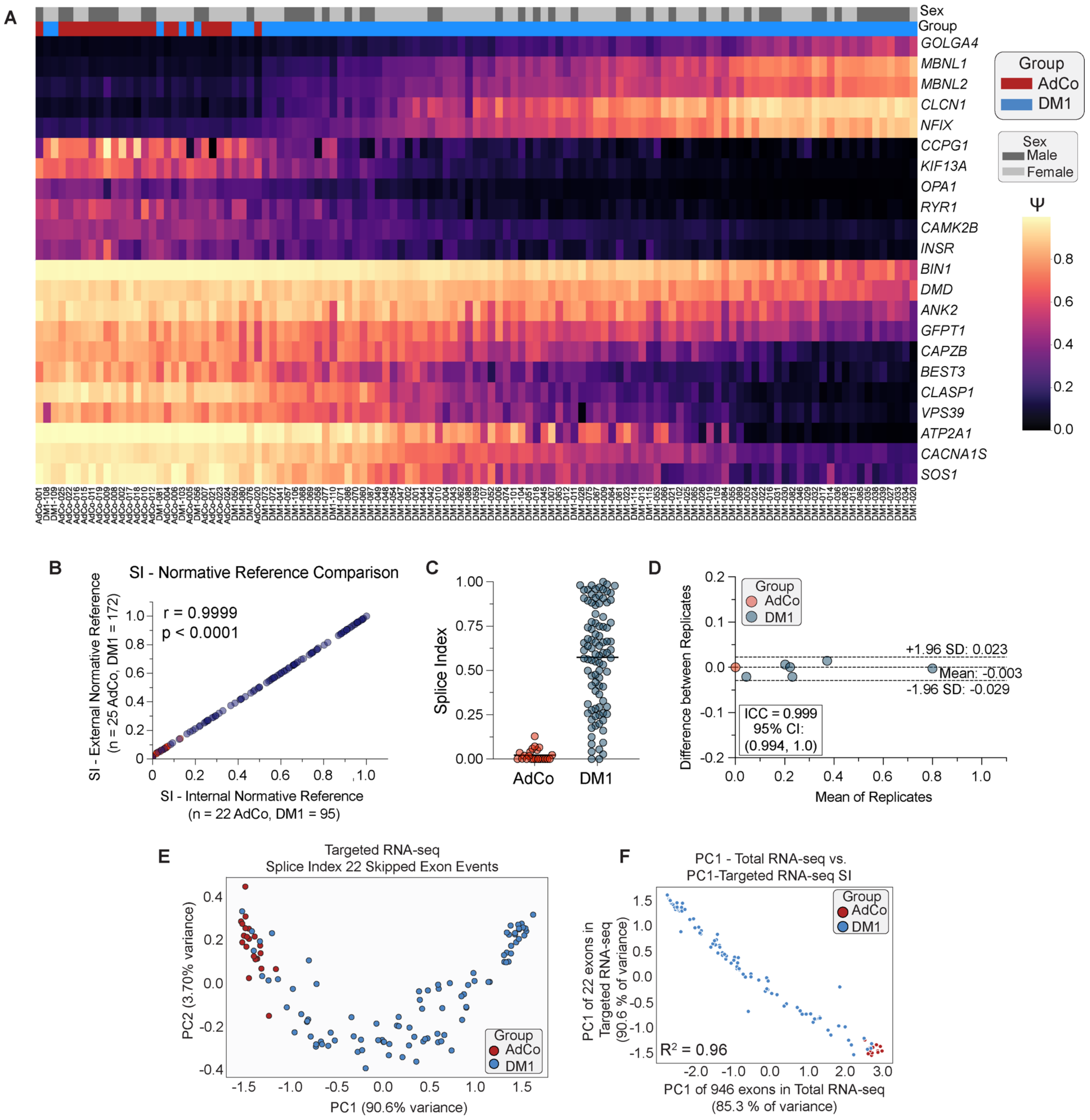

Supplemental Figure 4. Targeted RNA-seq of 22-events encompassing Splicing Index panel captures global spliceopathy in DM1 skeletal muscle. (A) Heatmap displaying normalized Ψ values for 22 SI splicing events derived from targeted RNA-seq of DM1 and AdCo samples subjected to targeted RNA-seq (Supplemental Table 5). Columns (individuals subjects) are force-ranked by SI from 0 to 1 while rows (splice events) were subjected to hierarchical clustering. Sample group and sex are annotated above and Subject ID is listed below. (**B**) Strong agreement is observed between both AdCo (n = 22) and DM1 (n = 95) SI scores derived using internal normative reference set (Supplemental Table 6) versus SI scores derived using external normative reference; Pearson *r* = 0.9999 [0.9998,0.9999], n = 117. (**C**) Distribution of SI scores in DM1 and AdCo subjects (n = 95 DM1, 22 AdCo). (**D**) Bland Altman plot illustrating agreement between SI scores derived from technical replications of 6 DM1 and 1 AdCo sample using targeted RNA-seq. Inter-rater reliability is reported as an ICC with 95% confidence intervals ICC = 0.999 [0.994, 1.0], p < 0.00001).(**E**) Principal component analysis of Ψ values for 22 splicing events encompassing the composite Splicing Index derived from targeted RNA-seq. (**F**) Correlation plot of principal component 1 from total RNA-seq (Supplemental Figure 1B) versus (**D**) targeted RNA-seq. R^2 is reported. All statistical analyses reported with [95% CI] with two tailed p-value, ****p < 0.0001

Supplemental Figure 5

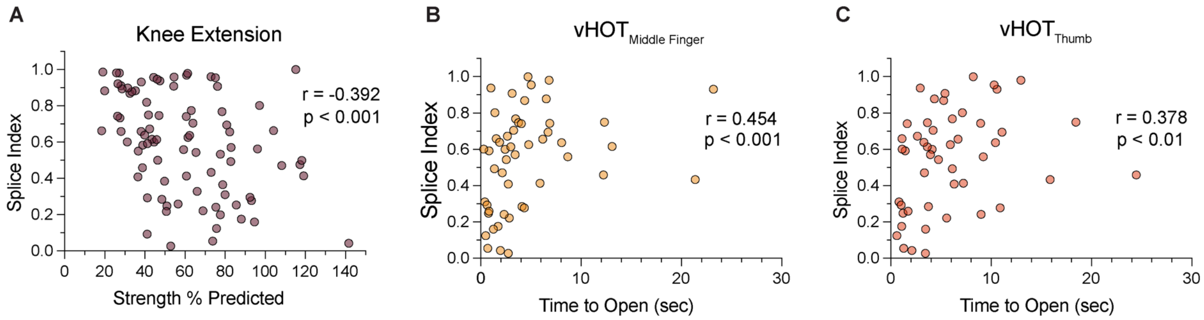

Supplemental Figure 5. Splicing Index correlates mildly with clinical outcome measures of proximal muscle strength and myotonia in cross-sectional DM1 cohort. (**A**) Correlation plot of SI versus quantitative measure of knee extension (KE) strength. Individual measures are reported as the percent of predicted strength compared to unaffected individuals. n = 87, Pearson *r* = -0.392 [-0.556, -0.197]. (**B** and **C**) Correlation plot of SI versus video hand opening time ($vHOT_{Middle\ Finger}$ and $vHOT_{Thumb}$). Individual measures are reported as time to open closed fist (seconds). $vHOT_{Middle\ Finger}$, n = 50, Spearman *r* = 0.454 [0.193, 0.655] & $vHOT_{Thumb}$, n = 49, Spearman *r* = 0.377 [0.099, 0.601]. All correlations are reported as Pearson/Spearman *r* [95% CI] with two-tailed p-value, ***p < 0.001, **p < 0.01.

Supplemental Figure 6

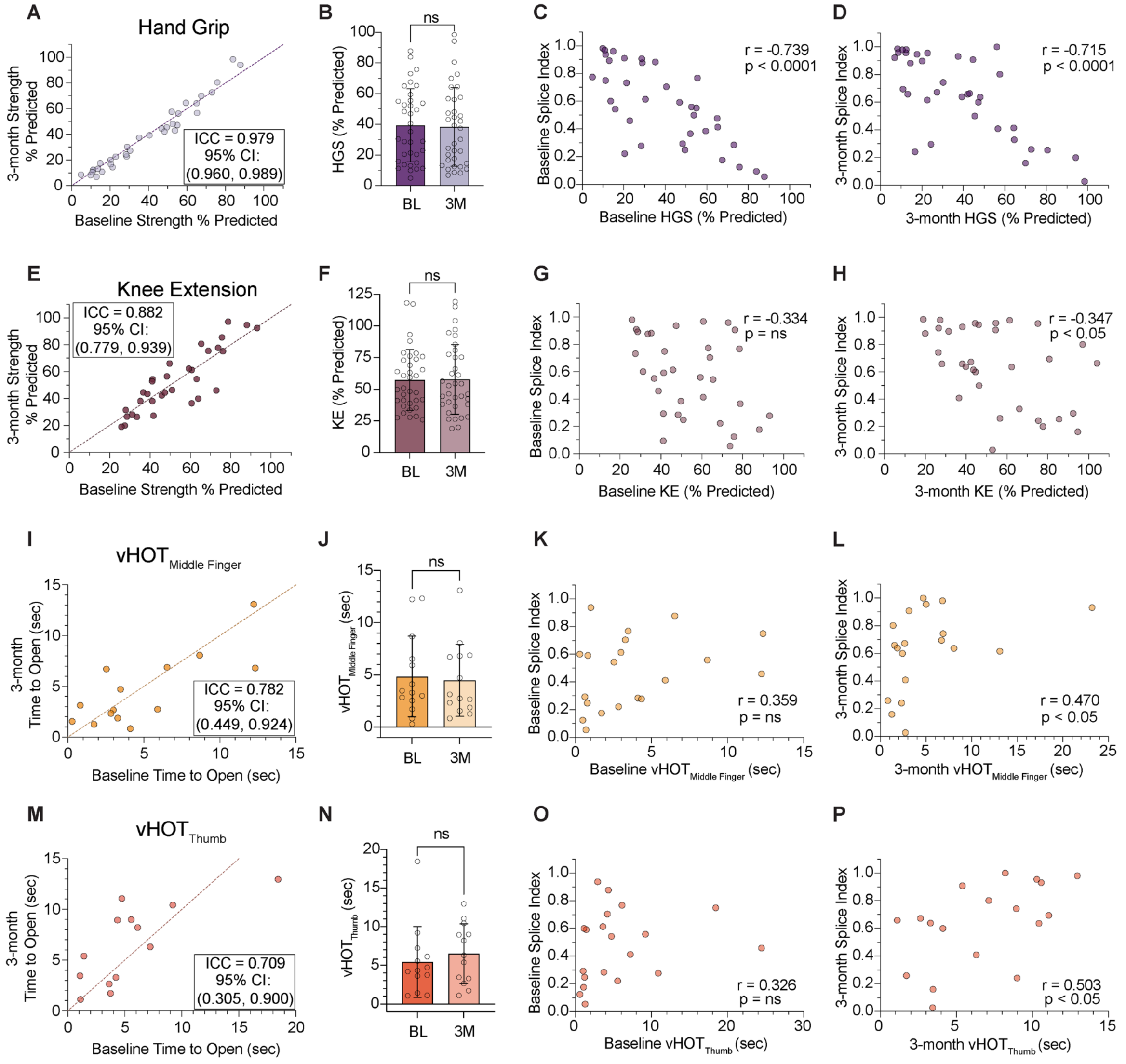

Supplemental Figure 6. Baseline and 3-month Splicing Index correlations to timepoint matched clinical outcome measures of muscle strength and myotonia in longitudinal DM1 sub-cohort.
(**A**, **E**, **I**, **M**) Assessment of changes in (**A**) hand grip strength (HGS), (**E**) knee extension strength (KE), (**I**) vHOT of the middle finger, and (**M**) vHOT of the thumb. Agreement between baseline (BL) and 3-month (3M) outcome measures in longitudinally sampled DM1 cohort is displayed with line of agreement (x = y) for reference. Test-retest reliability was evaluated with intraclass correlation coefficient (ICC) with 95% CI. n = 35, 35, 14, and 13, respectively (**B**, **F**, **J**, **N**) Mean performance is unchanged between BL and 3M in longitudinal DM1 cohort when evaluated using measures of (**B**) HGS, (**F**) KE, (**J**) $vHOT_{Middle\ Finger}$, and (**N**) $vHOT_{Thumb}$. Data presented as mean ± SD, n = 35 – 13; paired t-test, ns = not significant. (**C** and **D**, **G** and **H**, **K** and **L**, **O** and **P**) Correlations of baseline (**C**, **G**, **K**, **O**) and 3-month (**D**, **H**, **L**, **P**) Splice Index scores to timepoint matched measures of (**C** and **D**) HGS, (**G** and **H**) KE, (**K** and **L**) $vHOT_{Middle\ Finger}$, and (**O** and **P**) $vHOT_{Thumb}$. (**C** and **D**) BL SI v. BL HGS, n = 35, Pearson *r* = -0.739 [-0.860, -0.538]. 3M SI v. 3M HGS, n = 35 Pearson *r* = -0.715 [-0.846, -0.504]. (**G** and **H**) BL SI v. BL KE, n = 35, Pearson *r* = -0.334 [-0.600, -0.001] . 3M SI v. 3M KE, n = 35, Pearson *r* = -0.347 [-0.610, -0.016]. (**K** and **L**) BL SI v. BL $vHOT_{Middle\ Finger}$, n = 20, Spearman *r* = 0.359 [-0.113, 0.699]. 3M SI v. 3M $vHOT_{Middle\ Finger}$, n = 19 Spearman *r* = 0.470 [0.006,0.768]. (**O** and **P**) $vHOT_{Thumb}$ BL SI v. BL $vHOT_{Thumb}$, n = 20, Spearman *r* = 0.326 [-0.150, 0.680]. 3M SI v. 3M $vHOT_{Thumb}$, n = 18, Spearman *r* = 0.503 [0.032, 0.791]. HGS and KE measures are reported as the percent of predicted strength compared to unaffected individuals. vHOT measures are reported as the time to open closed fist (seconds). All correlations are reported as Pearson or Spearman r [95% CI] with two tailed p-value, ****p < 0.0001, *p < 0.05, ns = not significant.

Supplemental Figure 7

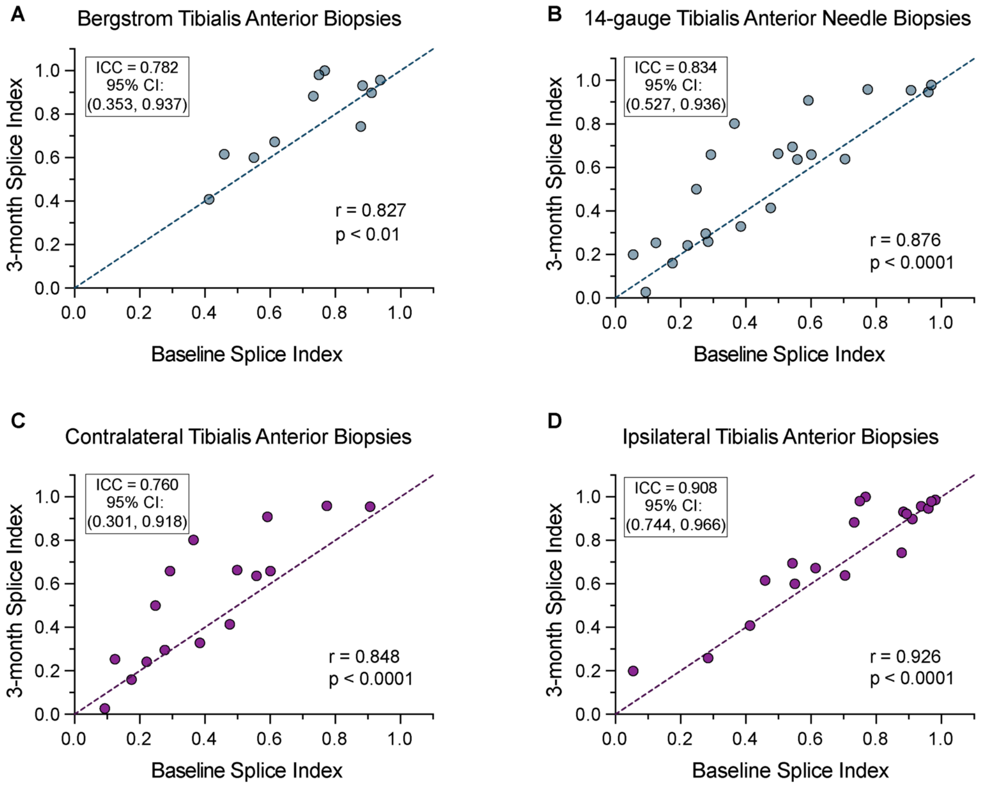

Supplemental Figure 7. Correlation of baseline and 3-month Splice Index values in longitudinally sampled DM1 participants is similar independent of biopsy type or leg used for repeat biopsy. (**A** and **B**) Assessment of change between baseline and 3-month Splice Index (SI) scores derived from tibialis anterior biopsies collected via (**A**) Bergstrom or (**B**) 14-guage needle biopsy. Agreement between SI scores is displayed with line of agreement (x = y). Test-retest reliability was evaluated with intraclass correlation coefficient (ICC) with 95% CI. (**A**) Bergstrom Pearson r = 0.827 [0.452, 0.952], n = 11 & (**B**) needle Pearson r = 0.876 [0.721, 0.948], n = 22. (**C** and **D**) Assessment of change between baseline and 3-month (SI) scores where 3-month biopsy was collected from (**C**) contralateral or (**D**) ipsilateral tibialis anterior muscle. (**C**) Contralateral Pearson r = 0.848 [0.607, 0.946], n = 16 & (**D**) ipsilateral Pearson r = 0.926 [0.813, 0.971], n = 19. All correlations are reported as Pearson r [95% CI] with two-tailed p-value, ****p < 0.0001, **p < 0.01.

Supplemental Figure 8

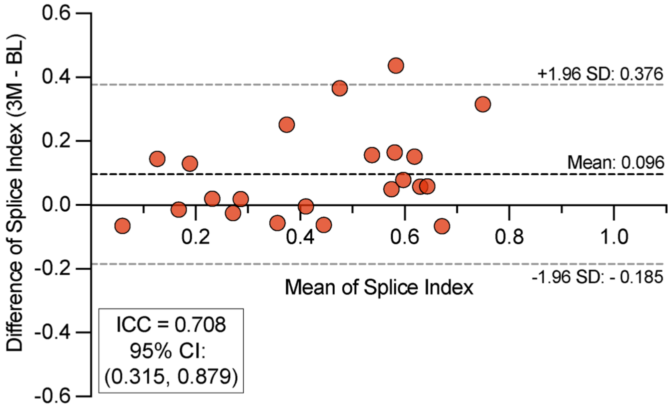

Supplemental Figure 8. Participants with high Splice Index values anchor relative stability of score between baseline and 3-months. Bland Altman plot illustrating agreement between Splicing Index scores at BL and 3M in DM1 longitudinal DM1 sub-cohort excluding individuals with high average SI scores (Mean SI > 0.8), n = 23. Dotted lines represent mean of the differences (bias) and 95% limits of agreement (mean of differences ± 1.96 SD). Intraclass correlation coefficient (ICC) with 95% CI is reported; $p < 0.0001$.

Supplemental Figure 9

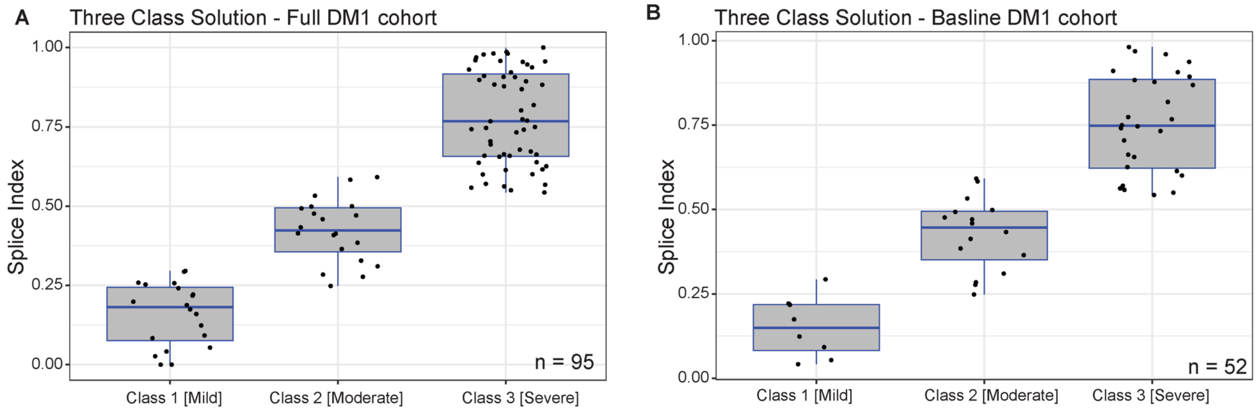

Supplemental Figure 9. Distribution of Splicing Index values by latent class assignment. Box and whisker plot of splicing index (SI) values in latent class assignments. Line represents median value and whiskers extend to minimum and maximum value. The points show actual participant SI values. Random noise has been added to the horizontal position (i.e., x-axis) but not the vertical position (i.e., y-axis) to reduce overplotting. Statistical analyses of each LCA model and comparison of class demographics and associated outcome measures are reported in Supplemental Table 8. (**A**) LCA class assignment derived using entire study cohort with available targeted RNA-seq data (n = 129). Only DM1 participants are plotted (n = 95). (**B**) LCA class assignment derived using DM1 participants with baseline functional assessments only (n = 52).

Supplemental Figure 10

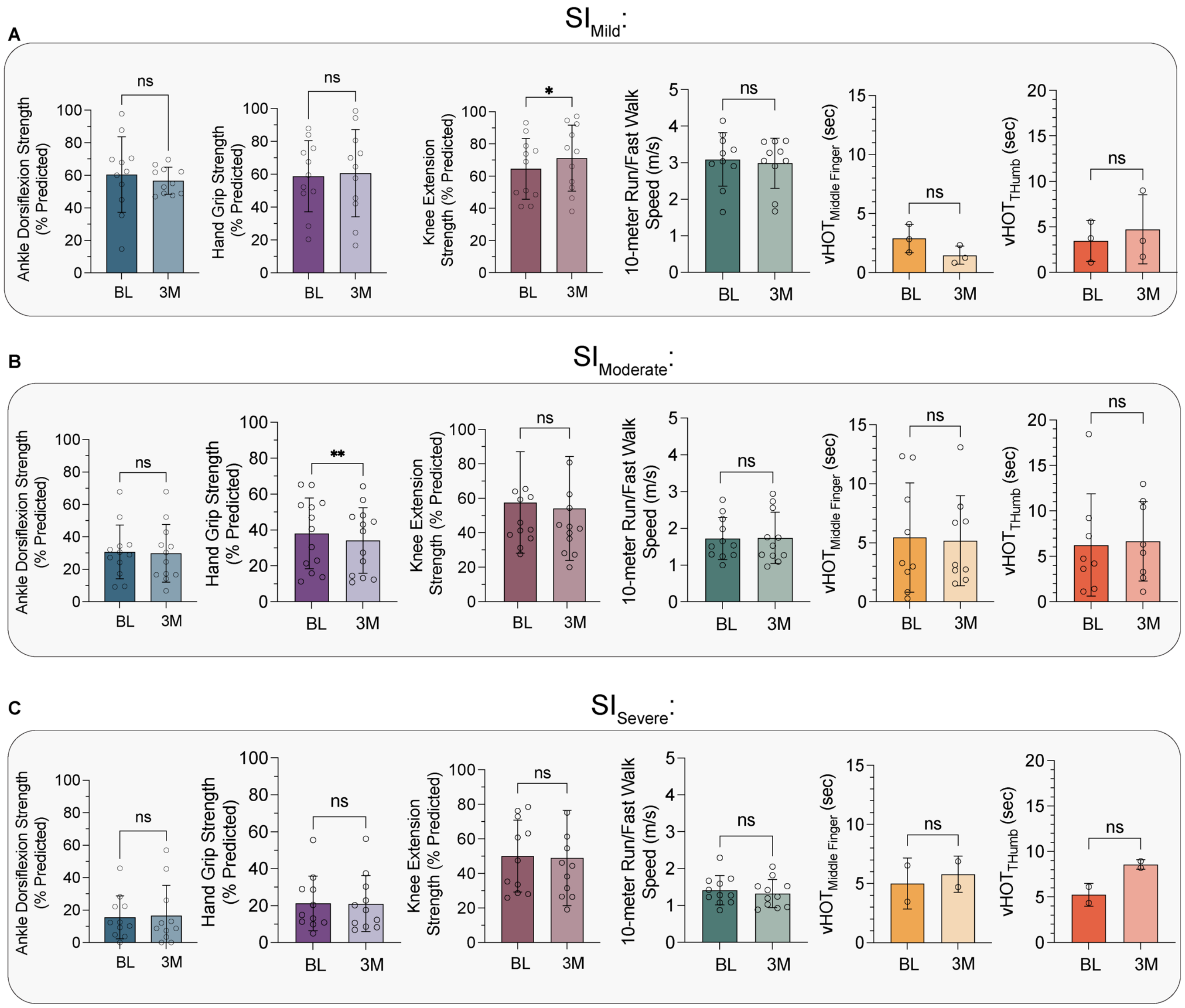

Supplemental Figure 10. Mean performance on muscle strength and functional motor assessments are unchanged between BL and 3M timepoints in Splice Index stratified sub-cohorts for nearly all clinical outcomes. (A – C) Average performance on measures of muscle strength (ankle dorsiflexion, hand grip, and knee extension), ambulation (10-meter run/fast walk), and myotonia ($vHOT_{Middle\ Finger}$, $vHOT_{Thumb}$) is unchanged between baseline (BL) and 3-month (3M) timepoints in (A) SI_{Mild}, (B) $SI_{Moderate}$, and (C) SI_{Severe} sub-cohorts. ADF, HGS, and KE measures are reported as the percent of predicted strength compared to unaffected individuals. 10MRW is reported as speed (meters/second). vHOT measures are reported as the time to open closed fist (seconds). Data represented as mean ± SD, n = 2 – 35 ; paired t-test, ns = not significant, *p < 0.05, **p < 0.001.

Supplemental Figure 11

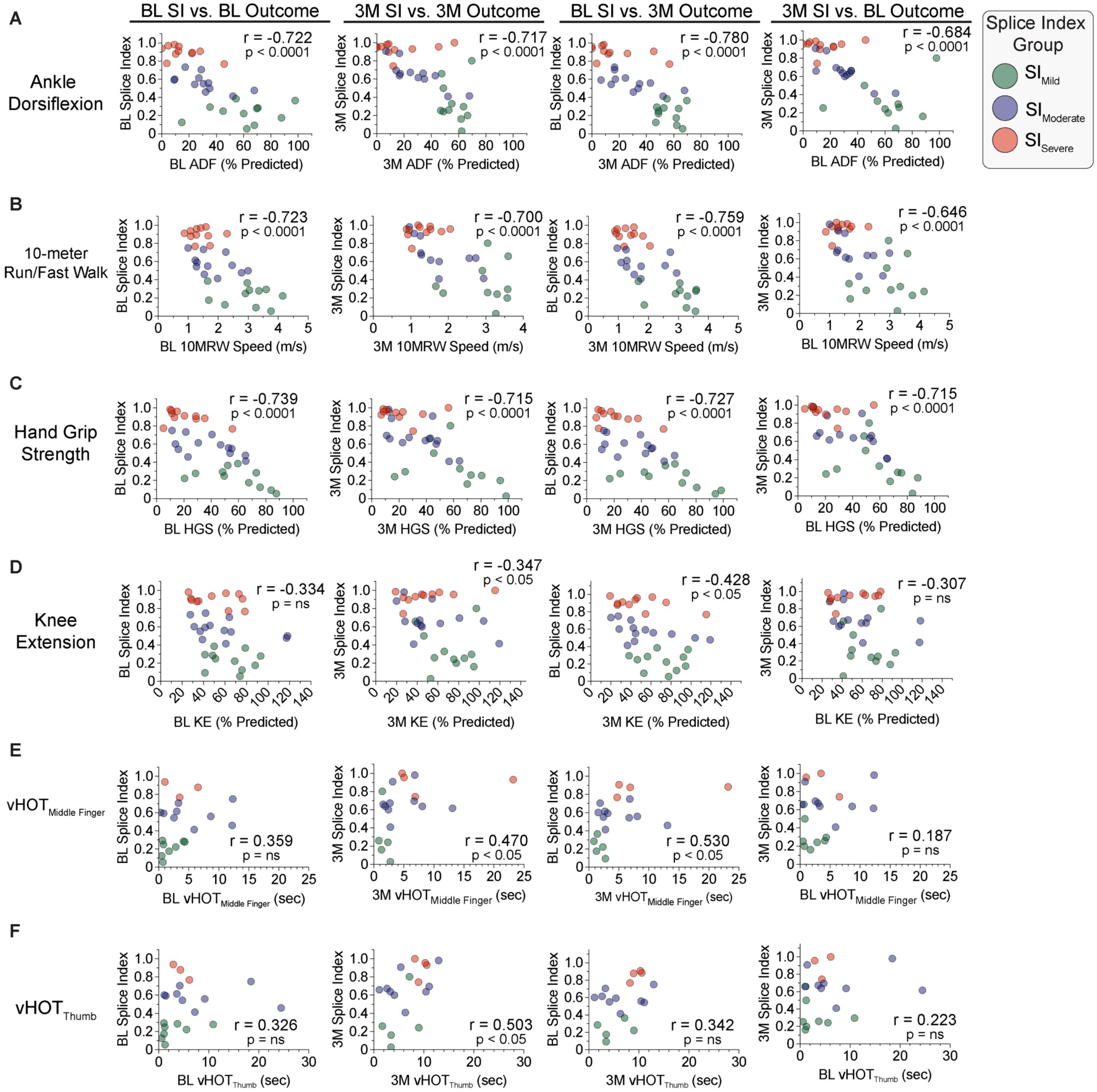

Supplemental Figure 11. Baseline Splice Index scores demonstrate an increased correlation with 3-month outcomes. (**A** – **F**) Correlation analysis of baseline (BL) and 3-month (3M) Splice Index (SI) scores to timepoint matched and mis-matched clinical outcome measures of skeletal muscle strength, ambulation, and myotonia. In each panel from left to right: BL SI v. BL measure, 3M SI v. 3M measure, BL SI v. 3M measure, and 3M SI v. BL measure, respectively. Individual samples are colored by SI_{Mild}, $SI_{Moderate}$, and SI_{Severe} groups. All correlations are reported as Pearson or Spearman r [95% CI] with two-tailed p-value; **** $p < 0.0001$, *$p<0.05$, ns = not significant. (**A**) Ankle dorsiflexion (ADF). Individual measures are reported as the percent of predicted strength compared to unaffected individuals (n = 34). BL SI v. BL ADF Pearson r = -0.722 [-0.852, -0.507], 3M SI v. 3M ADF Pearson r = -0.717 [-0.849, -0.499], BL SI v. 3M ADF Pearson r = -0.780 [-0.885, -0.600], and 3M SI v. BL ADF Pearson r = -0.684 [-0.830, -0.449]. (**B**) 10-meter run/fast walk (10MRW). Individual measures are reported as speed (m/s) (n = 32 – 34). BL SI v. BL 10MRW Pearson r = -0.725 [-0.854, -0.513], 3M SI v. 3M 10MRW Pearson r = -0.700 [-0.843, -0.465], BL SI v. 3M 10MRW Pearson r = -0.759 (95% CI = [-0.876, -0.559], and 3M SI v. BL 10MRW Pearson r = -0.646 [-0.809, -0.395]. (**C**) Hand grip strength (HGS). Individual measures are reported as the percent of predicted strength compared to unaffected individuals. BL SI v. BL HGS Pearson r = -0.739 [-0.860, -0.538], 3M SI v. 3M HGS Pearson r = -0.715 [-0.846,

-0.500], BL SI v. 3M HGS Pearson *r* = -0.727 [-0853, -0.519], and 3M SI v. BL HGS Pearson *r* = -0.715 [-0.847, -0.501]. (**D**) Knee extension strength (KE). Individual measures are reported as the percent of predicted strength compared to unaffected individuals (n= 35). BL SI v. BL KE Pearson *r* = -0.334 [-0.600, -0.001], 3M SI v. 3M KE Pearson *r* = -0.347 [-0.610, -0.016], BL SI v. 3M KE Pearson *r* = -0.428 [-0.666, -0.110], 3M SI v. BL KE Pearson *r* = -0.307 [-0.581, 0.030]. (**E**) Video hand opening time (vHOT) of the middle finger. Individual measures are reported as time to open closed fist (seconds) (n = 19 – 20). BL SI v. BL $vHOT_{Middle\ Finger}$ Spearman *r* = 0.359 [-0.113, 0.700], 3M SI v. 3M $vHOT_{Middle\ Finger}$ Spearman *r* = 0.470 [0.001, 0.768], BL SI v. 3M $vHOT_{Middle\ Finger}$ Spearman *r* = 0.530 0.085, 0.799], and 3M SI v. BL $vHOT_{Middle\ Finger}$ Spearman *r* = 0.187 [-0.292, 0.590]. (**F**) Video hand opening time (vHOT) of the thumb (n = 18 – 20). BL SI v. BL $vHOT_{Thumb}$ Spearman *r* = 0.326 [-0.1450, 0.680], 3M SI v. 3M $vHOT_{Thumb}$ Spearman *r* = 0.504 [0.032,0.791], BL SI v. 3M $vHOT_{Thumb}$ Spearman *r* = 0.342 [-0.164, 0.705], and 3M SI v. BL $vHOT_{Thumb}$ Spearman *r* = 0.223 [-0.257, 0.614].

Supplemental Figure 12

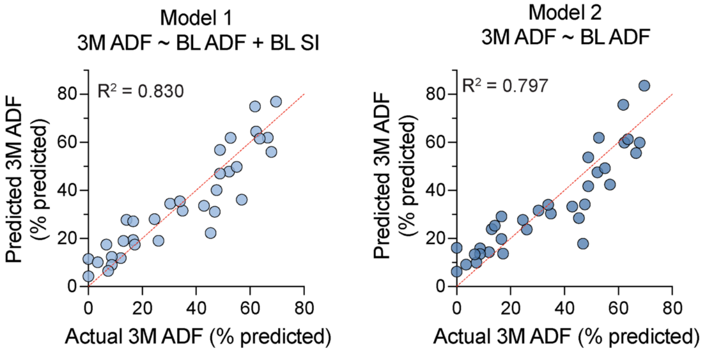

Supplemental Figure 12. Addition of baseline SI score with baseline ADF improves predictive power of multiple linear regression model to predict 3-month performance. Combination of baseline (BL) SI and timepoint matched measure of ankle dorsiflexion (ADF) strength (Model 1) is more predictive of performance at 3-months (3M) compared to BL ADF alone (Model 2). Agreement plot of actual versus predicted 3M ADF derived from multiple linear regression models. Quantitative parameters of models reported in complete intercept table provided in Supplemental Table 9.

Supplemental Figure 13

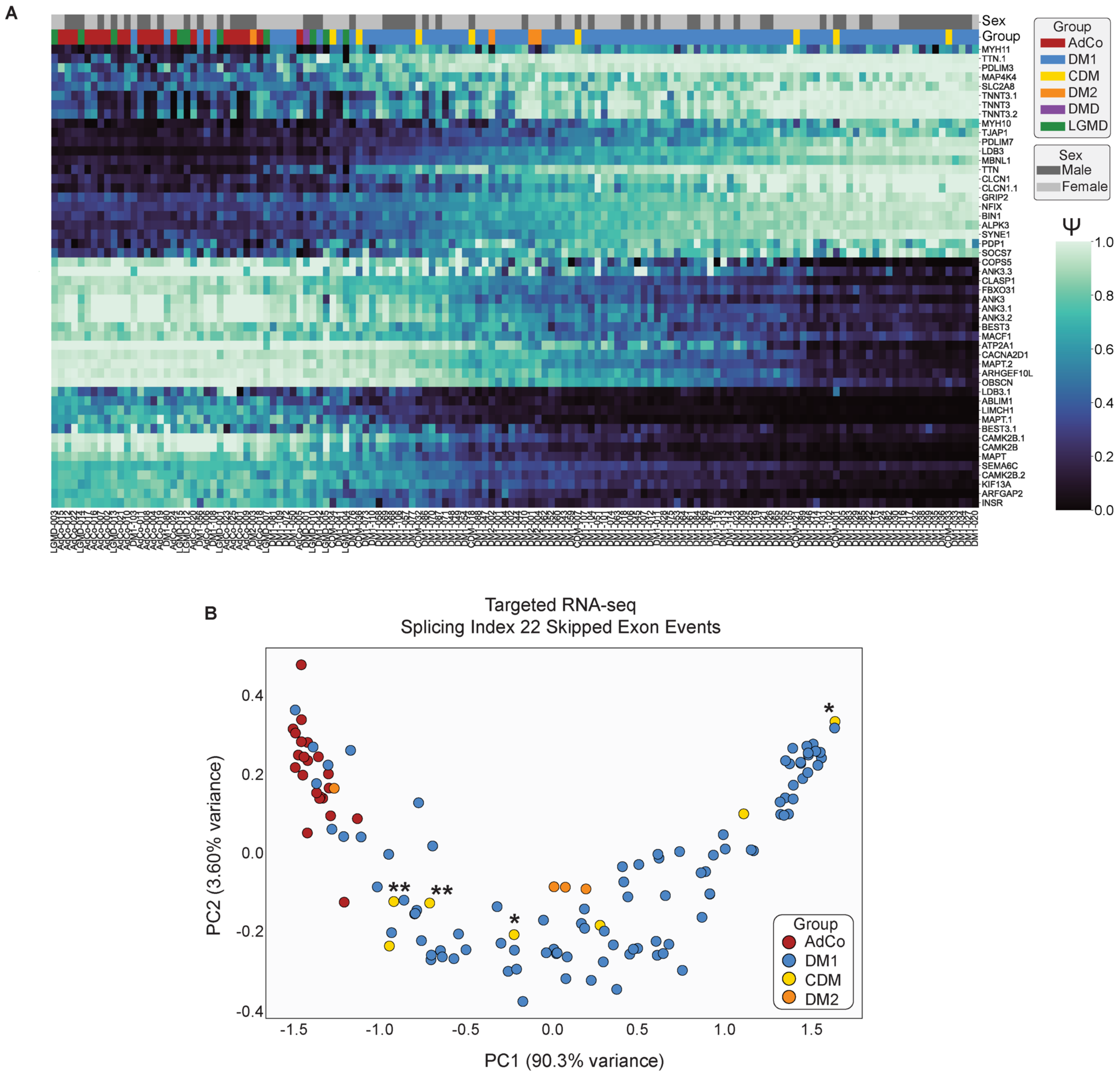

Supplemental Figure 13. Splice index (SI) distills splicing dysregulation in ambulatory DM2 subjects and longitudinally sampled CDM individuals. (**A**) Heatmap displaying estimated Ψ of top 50 significantly dysregulated skipped exon (SE) events between all DM participants (DM1, DM2, and CDM) versus unaffected adult controls (AdCo) & disease control reference groups (DMD & LGMD) subjected to total RNA-seq ($|\Delta\Psi| \geq 0.1$, FDR ≤ 0.05) (Supplemental Table 10). Both rows (SE events) and columns (individual samples) were subjected to hierarchical clustering. Sample group and sex are annotated above the heatmap and individual Subject IDs are reported below. (**B**) Use of SI RNA splicing events alone distributes CDM and DM2 participants within spread of DM1 splicing dysregulation. Principal component analysis of Ψ values for 22 splicing events encompassing the SI derived from targeted RNA-seq inclusive of DM2 and CDM samples. Ψ values utilized are reported in Supplemental Table 5. Longitudinally sampled CDM participants are annotated as *CDM-001 and CDM-018 sampled at 2 weeks and 8 years of age; **CDM-032 and CDM-036 sampled at 12 and 16 years of age. Repeat-biopsied CDM children show similar distribution within spread of DM1 and AdCo participants and demonstrate similar shifts between timepoints to that previously reported (1).

Supplemental Table Legends

Supplemental Table 1. Demographic table of DM1 participants and other subjects. DM1 Participant ID, Subject ID, sex, age at biopsy, disease group, biopsy method, muscle biopsied, and contralateral/ipsilateral biopsy collection are reported. For each participant, Splice Index scores are reported at each timepoint (baseline or 3-months). $[MBNL]_{inferred}$ as derived from total RNA-seq is also listed. Where available, clinical measures of muscle strength, motor function, and myotonia are provided. SRA accession numbers of associated total and targeted RNA-seq are listed.

Supplemental Table 2. Significantly dysregulated splicing events in DM1 skeletal muscle as determined by total RNA-seq. Ψ values of 946 skipped exon events defined as significantly dysregulated between DM1 subjects versus unaffected adult controls (AdCo) and disease controls (LGMD, DMD) ($|\Delta\Psi| \geq 0.1$, $FDR \leq 0.05$). Events are rank ordered by mean inclusion level difference and Ψ values for each individual sample are listed.

Supplemental Table 3. Ψ values and isoform counts of 22 skipped exon events encompassing Splicing Index panel derived from total RNA-seq. Ψ values for all DM1, AdCo, LGMD, and DMD subjects subjected to total RNA-seq. Skipped exon event coordinates, median Ψ & 95% CI for each sample group, and event inclusion & exclusion isoform counts per subject are reported.

Supplemental Table 4. Curve fitting parameters of early, intermediate, and late responder RNA splicing events included in Splicing Index panel. Parameters derived from curve fitting of total RNA-seq Ψ versus $[MBNL]_{inferred}$ using a four-parameter dose response curve (Figure 2) are listed, including Ψ_{min}, Ψ_{max}, span, slope, EC_{50}, and R^2 of fit. All values are reported as mean $\pm$ SEM.

Supplemental Table 5. Ψ values and isoform counts of 22 skipped exon events encompassing Splicing Index derived from targeted RNA-seq. Ψ for all DM1, AdCo, CDM, and DM2 subjects subjected to targeted RNA-seq of 22 event SI panel. Median Ψ & 95% CI for each sample group and event inclusion & exclusion isoform counts per subject are listed. Normalized Ψ values scaled using $\Psi_{Median\ Control}$ and Ψ_{DM95} reference values (Supplemental Table 6) are also reported.

Supplemental Table 6. Normative Ψ reference values derived for scaling of individual sample Ψ for 22 splice events within the composite Splicing Index. $\Psi_{Median\ Control}$ and Ψ_{DM95} reference values derived from 22 unaffected adult controls (AdCo) and all 95 DM1 samples subjected to targeted RNA-seq.

Supplemental Table 7. Individual RNA splice event associations with clinical outcome assessments in cross-sectional DM1 cohort. Individual correlations of normalized targeted RNA-seq Ψ (Supplemental Table 5) for 22 splice events encompassed within the composite Splicing Index to outcomes assessments, including ankle dorsiflexion (ADF), hand grip strength (HGS), knee extension (KE), 10-meter run/fast walk, $vHOT_{Thumb}$, and $vHOT_{Middle\ Finger}$. Number of matched samples (n), 95% CI, R^2, and p-value are reported for each splice event. Events are listed and force-ranked by Pearson *r*.

Supplemental Table 8. Latent class assignment model information and individual class correlations with functional outcome measures. Statistical measures of LCA models goodness of fit using two and three class solutions in entire study cohort with targeted RNA-seq data (n = 129) and DM1 subjects with baseline outcome measures (BL only, n = 52). Comparison of demographic variables (age, sex) and mean functional outcomes measures between LCA defined classes is also reported.

Supplemental Table 9. Complete intercept table of multiple linear regression Models 1 & 2. Companion table to regression models reported in Figure 7 & Supplemental Figure 12. Multiple linear regression analysis comparing two models using baseline Splice Index (BL SI) & baseline ADF (BL ADF) (Model 1) or BL ADF alone (Model 2) to predict ADF performance at 3-months (3M ADF). The analysis includes an examination of variance components, parameter estimates, and goodness-of-fit metrics. Multiple statistical elements are reported (sum of squares (SS), degrees of freedom (DF), mean squares (MS), F-statistic (F), standard errors (SE), 95% confidence intervals (CI), t-values, and p values.

Supplemental Table 10. Significantly dysregulated RNA splicing events in DM1, DM2, and CDM participant skeletal muscle as determined by total RNA-seq. Ψ values of the top 50 most significantly dysregulated skipped exon events between DM1, CDM, and DM2 subjects versus unaffected adult controls (AdCo) & disease controls (LGMD, DMD) ($|\Delta\Psi| \geq 0.1$, $FDR \leq 0.05$). Events are rank ordered by mean inclusion level difference and Ψ values for each individual sample are listed.

Supplemental Table 11. Splicing Index methodology reference materials. Table containing hg38 genome coordinates for 22 skipped exon events, multiplex PCR primers and cycling conditions, amplicon information and sequences, and custom reference FASTA sequence set for derivation of exon inclusion and exclusion counts.

References

1. Hale MA, et al. Dynamics and variability of transcriptomic dysregulation in congenital myotonic dystrophy during pediatric development. *Hum Mol Genet*. 2023;32(9):1413.

Supplemental Acknowledgements

DMCRN Consortium	
**Affiliation(s)**	**Role: Name, Titles**
University of Rochester Medical Center	**PI:** Johanna Hamel, MD<br>Associate Professor<br>Department of Neurology<br>Neuromuscular Disease and Department of Pathology and Laboratory Medicine<br><br>**Primary CRC:** Jeanne Dekdebrun, MS<br>Senior Research Coordinator<br><br>**Primary CE:** Katy Eichinger, PT, PhD, DPT, NCS<br>Associate Professor<br>Department of Neurology<br>Neuromuscular Division
The Ohio State University Wexner Medical Center<br><br>Department of Neurology, Division of Neuromuscular Diseases	**PI:** Bakri Elsheikh, MBBS, FRCP, FAAN<br>Professor of Neurology<br>Director of the Neuromuscular Division<br>Director of OSU Global Neurology Initiative<br>Director of the OSU Wexner Medical Center Muscular Dystrophy Association Care Center<br>Director of Clinical Neurophysiology Fellowship<br>Department of Neurology, Neuromuscular Division<br><br>**CRC:** Kaneshia Hives<br><br>**CE:** Kathryn Jira, PT, DPT
University of Kansas Medical Center	**PI:** Jeffrey Statland, MD<br><br>**Primary CRC:** Kaylene Whited, MS<br><br>**Primary CE:** Sandhya Sasidharan, PT
University of Florida College of Medicine	**PI:** Sub Subramony, MD<br>Professor of Neurology and Pediatrics<br>University of Florida College of Medicine<br><br>**Primary CRC:** Alex Rodriguez<br><br>**Primary CE**: Donovan Lott, PT, PhD, CSCS<br>Research Professor<br>Department of Physical Therapy
University of California, Los Angeles	**PI:** Perry Shieh, MD, PhD<br><br>**Primary CRC:** Dennis Fernando<br><br>**Primary CE:** Christy Skura, PT, DPT, PCS
Stanford University<br><br>Department of Neurology & Neurological Services	**PI:** Jacinda Sampson, MD, PhD<br><br>**Primary CRC:** Sara Ismail

	**Primary CE:** Tina Duong, PT, PhD
Houston Methodist Neurological Institute	**PI:** Ericka P. Greene, MD<br><br>**Primary CRC**: Aramide Balogun, BS<br><br>**Primary CE**: Dora Maldonado
University of Iowa	**PI:** Andrea Swenson, MD<br><br>**Primary CRC:** Maegan Tyrell, BA, CHES<br><br>**Primary CE:** Amy Yotty, PT, DPT<br>University of Iowa Health Care
Virginia Commonwealth University	**PI:** Nicholas Johnson, MD, M. Sci, FAAN<br><br>**Primary CRC:** Carino Jennings<br><br>**Primary CE:** Aileen Jones, PT, DPT
Radboud University Medical Center	**PI:** Karlien Mul, MD, PhD<br><br>**Primary CRC:** Monique Plieger<br><br>**Primary CE:** Judith van Engelen-Kanters
Fondazione Serena ONLUS - Centro Clinico NeMO Milano	**PI:** Valeria Sansone, MD, PhD<br><br>**Primary CRC:** Michela Nani, RN<br><br>**Primary CE:** Enrico Cossu, PT<br>Valentina Franchino, TNPEE
Friedrich Baur Institute, Department of Neurology, LMU University Hospital, LMU Munich	**PI:** Benedikt Schoser, FEAN<br><br>**Primary CRC/CE:** Corinna Wirner-Piotrowski, M Sc.
The National Hospital for Neurology and Neurosurgery, University College London Hospitals NHS Foundation Trust	**PI:** Chris Turner, MD, PhD, FRCP<br>Divisional Clinical Director, Queen Square.<br>Consultant Neurologist and UCL Honorary Clinical Associate Professor<br>University College Hospitals NHS Foundation Trust<br><br>**Primary CRC:** Nikoletta Nikolenko, MD, PhD<br><br>**Primary CE:** Charlotte Massey, BSc<br>Sheffield Institute for Translational Neuroscience (SITraN)<br>Department of Neuroscience, University of Sheffield
University of Colorado	**PI:** Thomas Ragole, MD<br>Assistant Professor<br><br>**Primary CRC**: Alyssa Avilez, BS<br><br>**Primary CE:** Talia Strahler, PT, DPT, OCS<br>Instructor
The University of Auckland, Centre for Brain Research Neurogenetics Clinic	**PI:** Richard Roxburgh, MBChB PhD FRACP<br><br>**Primary CRC**: Sarah Nagar, PGDipPH, BSc<br><br>**Primary CE:** Sarah Mollet, BHSc Physiotherapy<br>University of Auckland<br>CBR Neurogenetics Clinic
UC San Diego Health	**PI:** Chamindra Laverty, MD

Rady's Children's Hospital San Diego	**Primary CRC**: Mariah Stechschulte, BS<br><br>**Primary CE:** Kristine Negrete, DPT<br>Neurolab 360
Atkinson-Morley Neuromuscular Centre<br><br>St George's University Hospitals NHS Foundation Trust and Neuroscience and Cell Biology Research Institute<br><br>St George's University of London	**PI:** Emma Matthews, MBChB, PhD, FRCP<br><br>**Primary CRC:** Claire Gilmartin<br><br>**Primary CE:** Claire O'Farrell, BSc
Groupe de recherche interdisciplinaire sur les maladies neuromusculaires (GRIMN)<br><br>Integrated University Health and Social Services Centres (CIUSSS) of Saguenay Lac-St-Jean<br><br>Sherbrooke University	**PI:** Cynthia Gagnon, OT, PhD<br><br>**Primary CRC:** Justine Dolbec, M. Sc.<br><br>**Primary CE:** Amelie Lemire, B. Sc. Kin.
Osaka University	**PI:** Masanori Takahashi, MD, PhD<br>Osaka University<br><br>**Primary CRC:** Shizuko Taima, RN<br>Aomori National Hospital<br><br>**Primary CE:** Suzuki Manabu, RPT<br>Aomori National Hospital
University of Texas Health, Antonio	**PI:** Matthew Wicklund
